# Efficacy of decentralised home-based antihypertensive treatment in older adults with multimorbidity and polypharmacy (ATEMPT): an open-label randomised controlled pilot trial

**DOI:** 10.1016/S2666-7568(23)00259-3

**Published:** 2024-03

**Authors:** Jeannette Majert, Milad Nazarzadeh, Rema Ramakrishnan, Zeinab Bidel, Deborah Hedgecott, Abel Perez-Crespillo, Wendy Turpie, Naseem Akhtar, Moira Allison, Shishir Rao, Bernard Gudgin, Melanie McAuley, Christine A'Court, Laurent Billot, Dipak Kotecha, John Potter, Kazem Rahimi

**Affiliations:** a Deep Medicine, Oxford Martin School, Oxford, UK; b Nuffield Department of Women's and Reproductive Health, Medical Science Division, University of Oxford, Oxford, UK; c National Perinatal Epidemiology Unit, Nuffield Department of Population Health, University of Oxford, Oxford, UK; d Patient representatives ATEMPT, Oxford, UK; e Nuffield Department of Primary Health Sciences, University of Oxford, Oxford, UK; f The George Institute for Global Health, University of New South Wales, Sydney, NSW, Australia; g Institute of Cardiovascular Sciences, University of Birmingham, Birmingham, UK; h NIHR Birmingham Biomedical Research Centre & NHS West Midlands Secure Data Environment, University Hospitals Birmingham NHS Foundation Trust, Birmingham, UK; i Department of Medicine, University of East Anglia, Norwich, UK

## Abstract

**Background:**

Older patients with multimorbidity and polypharmacy have been under-represented in clinical trials. We aimed to assess the effect of different intensities of antihypertensive treatment on changes in blood pressure, major safety outcomes, and patient-reported outcomes in this population.

**Methods:**

ATEMPT was a decentralised, two-armed, parallel-group, open-label randomised controlled pilot trial conducted in the Thames Valley area, South East England. Individuals aged 65 years or older with multimorbidity (three or more chronic conditions) or polypharmacy (five or more types of medications) and a systolic blood pressure of 115–165 mm Hg were eligible for inclusion. Participants were identified through a search of national hospital discharge databases, identification of patients registered with an online pharmacy, and via targeted advertising on social media platforms. Participants were randomly assigned to receive up to two more classes versus up to two fewer classes of antihypertensive medications. Apart from routine home visits for conducting the baseline assessment, all communication, monitoring, and management of participants by the trial team was conducted remotely. The primary outcome was change in home-measured blood pressure.

**Findings:**

Between Dec 15, 2020, and Aug 31, 2022, 230 participants were randomly assigned (n=126 to more *vs* n=104 to fewer antihypertensive medications). The frequency of serious adverse events was similar across both groups; no cardiovascular events occurred in the more antihypertensive drugs group, compared with six in the fewer antihypertensive drugs group, of which two were fatal. Over a 13-month follow-up period, the mean systolic blood pressure in the group allocated to receive more antihypertensive medications decreased from 134·5 mm Hg (SD 10·7) at baseline to 122·1 mm Hg (10·5). By contrast, in the group allocated to receive fewer antihypertensive medications, it remained relatively unchanged, moving from 134·8 mm Hg (SD 11·2) at baseline to 132·9 mm Hg (15·3); this corresponded to a mean difference of –10·7 mm Hg (95% CI –17·5 to –4·0).

**Interpretation:**

Remotely delivered antihypertensive treatment substantially reduced systolic blood pressure in older adults who are often less represented in trials, with no increase in the risk of serious adverse events. The results of this trial will inform a larger clinical trial focusing on assessing major cardiovascular events, safety, physical functioning, and cognitive function that is currently in the planning stages. These results also underscore the efficiency of decentralised trial designs, which might be of broader interest in other settings.

**Funding:**

National Institute for Health Research Oxford Biomedical Research Centre and the Oxford Martin School.

## Introduction

Hypertension is one of the main risk factors for premature death and disability globally, and affects more than a billion individuals, resulting in an estimated 9·4 million deaths per year.^1^ Numerous clinical trials have shown that pharmacological blood pressure reduction effectively reduces the risk of cardiovascular events in at-risk populations and that the relative risk reduction afforded by antihypertensive treatment is proportional to the intensity of blood pressure reduction.^2^ However, due to restrictions in selection of participants in most clinical trials, the importance of blood pressure-lowering treatment in specific patient populations remains uncertain. One growing patient population in whom there is uncertainty about the effects of antihypertensive treatment is older patients with multimorbidity and polypharmacy, particularly those who only have mildly elevated blood pressure.^3^, ^4^ This uncertainty is also mirrored in inconsistent recommendations in clinical practice guidelines about the systolic blood pressure threshold for older patients.^5^, ^6^, ^7^ Although a systolic blood pressure target of less than 130 mm Hg is favoured for patients aged 65 years and older with multimorbidity by the American College of Cardiology and American Heart Association guideline,^5^ European guidelines recommend a range between 130 mm Hg and 139 mm Hg,^6^ and the National Institute for Health and Care Excellence in the UK suggests maintaining the target at 140–159 mm Hg.^7^

**Research in context**

**Evidence before this study**

We conducted a literature search on PubMed for trials published between Jan 1, 1966, and Aug 31, 2023. Search terms included “hypertension”, “antihypertensives”, “multimorbidity”, “polypharmacy”, “frailty”, and “randomised controlled trials”. Clinical trials have highlighted the effectiveness of blood pressure reduction in reducing cardiovascular risks overall, but uncertainties persist, particularly in older individuals with multiple health issues and on multiple medications who have normal or mildly elevated blood pressure. This uncertainty is reflected in inconsistent recommendations for systolic blood pressure thresholds. The limitation of evidence in this area arises from challenges in recruiting older patients with multimorbidity for clinical trials due to strict inclusion criteria, logistical issues, and concerns about short-term side-effects.

**Added value of this study**

The ATEMPT trial successfully achieved a substantial reduction in systolic blood pressure, from 134·5 mm Hg to 122·1 mm Hg in the intervention group. With an average difference of one antihypertensive drug between groups, a difference of 11 mm Hg in systolic blood pressure was achieved. This intervention did not significantly affect participants' quality of life, cognitive function, medication adherence, or frailty status, although there were reports of increased dizziness and fatigue with more antihypertensive drugs. The trial showed the feasibility and acceptability of remote medication delivery.

**Implications of all the available evidence**

The decentralised trial design proved effective in engaging older individuals with multiple health conditions and medication regimens, allowing them to participate from the comfort of their homes. The intervention successfully achieved a significant decrease in systolic blood pressure without adversely affecting participants' quality of life or cognitive function. These findings provide some reassurance about intensive blood pressure lowering in this under-represented patient group and could help inform the design of future studies.

One major cause for the substantial gap in the evidence of the effects of blood pressure treatment and the optimal target threshold is the challenge of recruiting a sufficient number of older patients with multimorbidity into clinical trials.^8^ A systematic review of phase 3 clinical trials conducted between 1965 and 2015 showed that patients were either explicitly excluded by age or did not pass the eligibility criteria due to the presence of several comorbidities, concomitant medications, or cardiac conditions.^8^ Apart from the restrictive inclusion criteria, another challenge is the burdensome trial procedures that often require regular travel to study clinics, which can lead to logistical difficulties for older patients with multimorbidity.^9^ With regard to blood pressure-lowering treatment, another difficulty is the concern about short-term side-effects of the treatment, which might not be captured appropriately in episodic clinic assessments. This concern is of particular importance in older patients with multimorbidity, given their altered drug metabolism, which could lead to an exaggerated and fluctuating response to treatment.^10^

A potential way to overcome the challenges of participant recruitment, monitoring, and follow-up in the growing population of older patients with multimorbidity is to design and conduct patient-centred home-based trials. The promise of this strategy was recently shown in a clinical trial of patients with heart failure and multimorbidity who, despite having little or no experience in using digital technologies, were successfully supported to use a tablet computer with a bespoke study application at home.^11^, ^12^ The remote communication system used in the trial was found to achieve high acceptability and satisfaction rates among older patients with multimorbidity while reducing the burden of monitoring on participants and study staff.^12^, ^13^

Thus, the Antihypertensive Treatment Evaluation in Multimorbidity and Polypharmacy Trial (ATEMPT) was designed to test the effectiveness of a similar decentralised home-based approach with little direct physical contact between participants and the study team. We aimed to investigate whether a substantial change in blood pressure can be achieved remotely in older patients with multimorbidity and average blood pressure readings without having any detrimental effects on safety or tolerability.

## Methods

### Study design and participants

ATEMPT was a decentralised, two-armed, parallel-group, open-label, randomised controlled pilot trial led by the University of Oxford (Oxford, UK). The trial was overseen by an independent trial steering committee, including lay members, to guide the research agenda, advise on the plan of investigation, and monitor the execution of the project on behalf of the sponsor and project funder. A data monitoring committee (DMC) was responsible for monitoring the trial data and the continued safety of research participants, with permission to access unblinded comparative data during the trial. Safety reviews of already collected trial data by the DMC were conducted on Nov 12, 2021, and Sept 12, 2022. The final version of the trial protocol (version 3.0 issued on April 26, 2021) is provided in the appendix (pp 14–15). There was one approved amendment to the protocol on April 26, 2021, which included changes to the trial team and steering committee members as well as updated contact details of the trial team (appendix p 40).

Participants living in the Thames Valley area (a region in South East England, which is centred on the River Thames west of London, with Oxford as its major centre), UK, were recruited and screened for eligibility. Potentially eligible participants were identified via three main routes: through a search of national hospital discharge databases, identification of patients registered with an online pharmacy, and via targeted advertising on social media platforms. The inclusion criteria comprised patients aged 65 years or older with multimorbidity (three or more underlying chronic conditions) or polypharmacy (taking five or more types of non-antihypertensive medications) and with a systolic blood pressure of 115–165 mm Hg. Comorbid conditions were defined as long-term medical conditions for which patients received active medical treatment or follow-up throughout the trial. Further inclusion criteria were participants' willingness to monitor their blood pressure at home and their or their carer's ability to use the web-based trial system. Patients with a history of admission to hospital with heart failure or known systolic heart failure or self-reported orthostatic hypotension were excluded. Complete inclusion and exclusion criteria are provided in the appendix (pp 14–15). Data on the ethnicity of participants were not collected.

The entire study workflow, data, and participant management as well as safety and clinical monitoring were implemented in a modular clinical trial management platform, Zeesta. Potential participants were invited by post or digitally via social media advertisement to log into Zeesta's online personal participant portal to learn more about the study via an interactive participant information sheet including a video infographic, to self-screen their eligibility, and to provide electronic informed consent (e-consent). Participants unsure of or unwilling to use the online portal could nominate a friend or carer to assist them with accessing and using the website during the registration process and throughout the study period if required. Additionally, a free telephone line and email address were available if participants or their carers preferred to contact the study team directly for further information.

Ethics approval (reference number 20/NW/0344) was obtained from the Greater Manchester South Research Ethics Committee before trial commencement.

### Run-in period

Participants who were screened and provided e-consent entered a run-in phase to assess their full eligibility before randomisation. This run-in phase involved a home visit to provide an upper arm cuff-based blood pressure monitor (A&D model UA-651BLE or UA-767 Plus BT-Ci, A&D Company, Tokyo, Japan), unless participants wished to use their own validated device, and to collect further information about participants' demographics, medical conditions, and treatment. The baseline assessment also included evaluation of participants' frailty status with the PRISMA-7 questionnaire,^14^ quality-of-life index with the EQ-5D-5L,^15^ cognitive function assessment with the telephone-based Montreal Cognitive Assessment (T-MoCA),^16^ and a medication adherence assessment. Participants were asked to measure their blood pressure and pulse once a day during the run-in phase (and once weekly afterwards) and to submit these measurements on Zeesta's participant portal. The process of taking blood pressure measurements was standardised by advising patients to measure their blood pressure at the same time of the day and to rest for at least 5 min in a seated position before taking the measurement. A mean value of all day-time measurements over a week was automatically calculated by Zeesta and served as the baseline pre-randomisation home blood pressure. To estimate clinic blood pressure values for eligibility assessment, 5 mm Hg was added to mean home blood pressure values.^7^

During the baseline home visit, blood samples were taken to check participants' renal function and electrolytes. Further blood analyses were conducted after randomisation according to participants' treatment allocation and treatment regimen throughout the trial.

### Randomisation and masking

A difference of two antihypertensive drugs between treatment groups was targeted, with an expected 10 mm Hg difference in systolic blood pressure.^17^ Aiming for a fixed difference in the intensity of blood pressure-lowering treatment has the advantage that participants with a wide range of pre-randomisation blood pressure measurements and antihypertensive medication use could be included. This approach also obviated the need for a single blood pressure target, which would be difficult to achieve across all baseline blood pressure groups, and enabled reliable testing of the study hypothesis.

Eligible participants were assigned to receive either more antihypertensive drugs or fewer antihypertensive drugs through the trial's electronic concealed randomisation system in Zeesta. Randomisation was based on a dynamic biased-coin minimisation algorithm with the categories of participant's age (≤80 years *vs* >80 years) and baseline clinic systolic blood pressure (<130 mm Hg, 130–140 mm Hg, and >140 mm Hg). Once the imbalances of these factors were estimated as a score, each participant was allocated to the group with the lowest score using a probability greater than 0·75. This method was used to dynamically minimise the imbalance between the groups. Once all relevant information had been collected, Zeesta triggered an alert to authorised central trial staff to perform the randomisation. A full audit trail of actions, including the randomisation seed, was recorded by Zeesta.

Depending on the number of antihypertensive medications and the systolic blood pressure at baseline, Zeesta then automatically divided participants in each randomised group into three strata to guide the treatment implementation, aiming for a minimum difference of two drug classes between randomised groups (table 1, appendix p 8). The trial management system restricted access to treatment allocation as per the protocol and according to user roles.

**Table 1:** Randomisation and stratum allocation

	**More antihypertensive medications**	**Fewer antihypertensive medications**
Stratum 1	Add two new drugs	No change
Stratum 2	Add one new drug	Stop one drug
Stratum 3	No change	Stop two drugs

The selection of antihypertensive medications followed the recommended order of the European Society of Hypertension guidelines.^6^ The antihypertensive agents were provided at no cost to participants and were directly delivered to their homes through the services provided by an online pharmacy. Drugs could be added at each assessment point every 4 weeks, after checking for known intolerances, drug interactions, and contraindications for those assigned to receive more hypertensive drugs. A maximum of one up-titration for each newly allocated drug with the aim of achieving half the daily recommended dose was targeted.^18^, ^19^ For those participants for whom deprescribing was recommended, one drug was reduced in dose or removed at each assessment point following the reverse order of guideline recommendations. Any antihypertensive agents prescribed for other compelling indications, such as atrial fibrillation, were not reduced or discontinued. Participants' general practitioners (GPs) were kept informed about any treatment changes, either reduction or intensification, initiated by the trial team throughout the study and were asked to update participants' repeat prescription lists.

### Procedures

The trial did not involve routine clinic assessments so all communication, monitoring, and management of participants by the trial team was conducted remotely; where necessary, home visits by a trained clinician were arranged. To examine the effect of changes to medication, participants' blood pressure values and treatment changes were reviewed remotely by a clinician and adjusted accordingly in 4-weekly intervals. Participants were encouraged to report any changes in their wellbeing or adverse events at any point during the follow-up period via Zeesta's participant portal or directly to the trial team through a freephone line. Additionally, changes to medication were automatically retrieved from record changes held by the online pharmacy, which was electronically linked to Zeesta. Every 3–6 months, a telephone call assessment was conducted by masked trial personnel for further treatment review, completion of quality of life, medication adherence, cognitive function, and frailty questionnaires as well as for reporting of any adverse events. The overall follow-up period was 13 months.

### Outcomes

The primary outcome of this pilot study was the change in remotely measured blood pressure. Secondary outcomes were the acceptability and tolerability of the remotely delivered intervention, assessed via patient-reported outcomes, and monitoring of adverse events. Additionally, cognitive function was measured with T-MoCA, frailty status was assessed with the PRISMA-7 questionnaire, and health-related quality of life was measured with the EQ-5D-5L. Participants' medication adherence was self-reported with a designed tool to check how they had been taking their antihypertensive medictions over the past 2 weeks. The trial also aimed to assess the feasibility of a planned large-scale trial. This assessment included the identification and recruitment of participants, remote monitoring and follow-up procedures, and evaluation of the resources needed for the pilot trial.

Occurrences of myocardial infarction, stroke or transient ischaemic attack, and heart failure or vascular procedures were investigated at each assessment point. Similarly, all serious adverse events and adverse events of interest (such as falls, fractures, dizziness, or confusion) were captured at each assessment point. Participants were further encouraged to report any other events that they felt might be related to any changes in antihypertensive treatment via the participant portal. User experience and treatment adherence were also investigated, as described above. In a survey that was conducted at the end of the follow-up period, participants were asked about their experience with the decentralised trial approach involving minimal physical contact with the trial team. The survey responses were assessed on a Likert scale with the following response options: 1=very dissatisfied, 2=dissatisfied, 3=neither satisfied or dissatisfied, 4=satisfied, and 5=very satisfied. For each question, participants also had the option to provide additional feedback that was captured as free text.

### Statistical analysis

Assuming an SD of 20 mm Hg in systolic blood pressure, a total sample size of 200 patients was calculated to provide 80% power to detect a mean difference of at least 8 mm Hg, and 90% power for a mean difference of at least 10 mm Hg. The mean change in systolic blood pressure from baseline to end of follow-up between the treatment groups was estimated with an independent sample *t*-test for each month. To plot the trajectory of change in systolic blood pressure and the count of antihypertensive drug classes during the follow-up time and between the two treatment groups, a linear mixed-effects modelling approach was used to take account of the irregularly spaced timepoints or data missingness at certain timepoints. The model included a random intercept and slope for time. The fixed effects included interaction of treatment and time and cubic effect of time, with an unstructured covariance structure of the model. For the secondary outcomes, descriptive statistics were computed with mean values for normal distributed outcomes, median values for skewed continuous outcomes, and counts and percentages for categorical outcomes. All analyses were done with R statistical software (version 3.4.4) and IBM SPSS Statistics (version 29.0.1.0).

To monitor safety aspects and the overall progress of the trial, interim analyses were conducted on Nov 12, 2021, and Sept 12, 2022, that were monitored by the DMC. Before each planned DMC meeting, an interim database lock was performed, and the trial statistician obtained blinded trial data for analysis. The preparation of the DMC reports was done to an agreed standard analysis and reporting format developed by the trial statistician with the support of the trial team and under the direction of the DMC. The reports were shared with the DMC members at least 5 days before scheduled meetings with a password-protected file. Operational bias was minimised by keeping trial statisticians masked to participants' treatment allocation and by the oversight of independent DMC members.

The trial is registered with ISRCTN (ISRCTN17647940).

### Role of the funding source

The funders of the study had no role in study design, data collection, data analysis, data interpretation, writing of the report, or the decision to submit the manuscript for publication.

## Results

**Figure 1:**
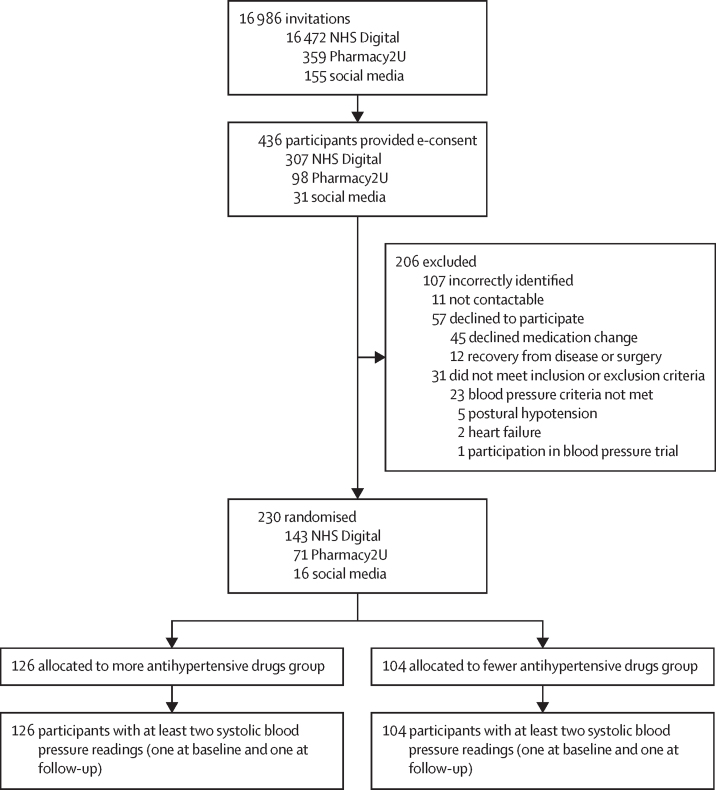
Trial profile No participants were lost to follow-up. In the fewer antihypertensives group, six participants withdrew from the trial and two died. In the more antihypertensives group, four participants withdrew from the study and one died. NHS=UK National Health Service. e-consent=electronic consent.

Between Dec 15, 2020, and Aug 31, 2022, 436 participants gave their consent for participation. Of these, 206 were ineligible; 230 were therefore randomly assigned (n=126 to receive more *vs* n=104 to receive fewer antihypertensive medications; figure 1). Taking account of pre-randomisation systolic blood pressure values and the number of antihypertensive classes that could potentially be stopped, the randomly assigned participants were further divided into three strata, aiming for a difference of two drug classes in each stratum. 136 (59%) of 230 participants were allocated to stratum 1 (two drug classes added *vs* no change), 75 (33%) of 230 were allocated to stratum 2 (one drug class added *vs* one drug class stopped), and 19 (8%) of 230 were allocated to stratum 3 (no change *vs* two drug classes stopped).

The characteristics of study participants were well balanced between the two treatment groups (table 2). The mean age of participants was 76·0 years (SD 6·1), and 118 (51%) of 230 participants were women. The mean systolic blood pressure was 134·5 mm Hg (SD 10·7). On average, each participant had a history of taking 1·5 (SD 1·1) non-trial antihypertensive drugs at baseline. Participants were, on average, on 5·7 (SD 2·4) non-antihypertensive classes of drugs and 184 (80%) of 230 had three to five comorbidities (excluding hypertension). The quality-of-life indices (appendix p 10), cognitive function (appendix p 9), frailty score, and medication compliance (appendix pp 4–5) were comparable between both groups at baseline.

**Table 2:** Baseline characteristics of analysed participants

		**Total (n=230)**	**More antihypertensive medications (n=126)**	**Fewer antihypertensive medications (n=104)**
Age, years		76·0 (6·1)	75·8 (6·3)	76·2 (6·0)
Systolic blood pressure, mm Hg		134·5 (10·7)	134·3 (10·2)	134·8 (11·2)
Age categories				
	≤80 years	177 (77%)	95 (75%)	82 (79%)
	>80 years	53 (23%)	31 (25%)	22 (21%)
Systolic blood pressure categories				
	<130 mm Hg	86 (37%)	49 (39%)	37 (36%)
	130–140 mm Hg	77 (33%)	42 (33%)	35 (34%)
	>140 mm Hg	67 (29%)	35 (28%)	32 (31%)
Sex				
	Men	111 (48%)	67 (53%)	44 (42%)
	Women	118 (51%)	59 (47%)	59 (57%)
	Missing	1 (1%)	0	1 (1%)
Treatment stratum after randomisation				
	Stratum 1: increase of two drugs *vs* no change	136 (59%)	80 (63%)	56 (54%)
	Stratum 2: increase *vs* reduction of one drug	75 (33%)	37 (29%)	38 (37%)
	Stratum 3: no change *vs* reduction of two drugs	19 (8%)	9 (7%)	10 (10%)
Number of antihypertensive medications		1·5 (1·1)	1·5 (1·1)	1·5 (1·1)
Antihypertensive drug class				
	None	42 (18%)	23 (18%)	19 (18%)
	ACE inhibitors	62 (27%)	39 (31%)	23 (22%)
	ARBs	66 (29%)	31 (25%)	35 (34%)
	Beta-blockers	56 (24%)	34 (27%)	22 (21%)
	Calcium-channel blockers	96 (42%)	50 (40%)	46 (44%)
	Diuretics	48 (21%)	24 (19%)	24 (23%)
	Alpha1-receptor blockers	18 (8%)	9 (7%)	9 (9%)
	Potassium-sparing diuretics	2 (1%)	1 (1%)	1 (1%)
Dose of antihypertensive drugs*				
	ACE inhibitors	57 (25%)	37 (29%)	20 (19%)
	ARBs	50 (22%)	21 (17%)	29 (28%)
	Beta-blockers	24 (10%)	16 (13%)	8 (8%)
	Calcium-channel blockers	88 (38%)	46 (37%)	42 (40%)
	Diuretics	38 (17%)	19 (15%)	19 (18%)
	Alpha1-receptor blockers	17 (7%)	8 (6%)	9 (9%)
	Potassium-sparing diuretics	1 (<1%)	0	1 (1%)
Number of non-antihypertensive medications		5·7 (2·4)	5·7 (2·5)	5·8 (2·3)
Comorbid conditions (not including hypertension)				
	≤2	11 (5%)	5 (4%)	6 (6%)
	3–5	184 (80%)	99 (79%)	85 (82%)
	>5	35 (15%)	22 (17%)	13 (13%)
Comorbid diseases				
	Stroke	8 (3%)	4 (3%)	4 (4%)
	Coronary heart disease	42 (18%)	30 (24%)	12 (12%)
	Diabetes	45 (20%)	17 (13%)	28 (27%)
	Chronic kidney disease	20 (9%)	11 (9%)	9 (9%)
	Atrial fibrillation	42 (18%)	26 (21%)	16 (15%)
EQ-5D-5L score				
	Health state index	7·8 (2·6)	7·6 (2·6)	7·9 (2·6)
	VAS (perceived health status)	78·0 (13·9)	78·1 (13·2)	77·9 (14·7)
	EQ-5D value set	0·7 (0·1)	0·7 (0·1)	0·7 (0·1)
T-MoCA score (cognitive function)		19·5 (2·0)	19·7 (1·8)	19·3 (2·2)
Cognitive impairment		59 (26%)	28 (22%)	30 (29%)
PRISMA frailty scale		2·7 (1·6)	2·6 (1·4)	2·8 (1·7)
Drug compliance		4·2 (1·5)	4·2 (1·5)	4·1 (1·6)
Laboratory measurements				
	Blood urea, mmol/L	7·6 (7·5)	7·3 (3·9)	7·9 (9·7)
	Serum creatinine, μmol/L	86·2 (31·4)	86·1 (32·2)	86·3 (30·9)
	Serum sodium, mmol/L	139·6 (2·8)	140·1 (2·1)	139·2 (3·2)
	Serum potassium, mmol	4·8 (0·5)	4·7 (0·5)	4·8 (0·5)

Data are n (%) or mean (SD). ACE=angiotensin-converting enzyme. ARB=angiotensin receptor blocker. VAS=Visual Analogue Scale. T-MoCA=Telephone Montreal Cognitive Assessment.

* Number (%) of participants who met the minimal dose recommended by the British National Formulary (BNF).

**Figure 2:**
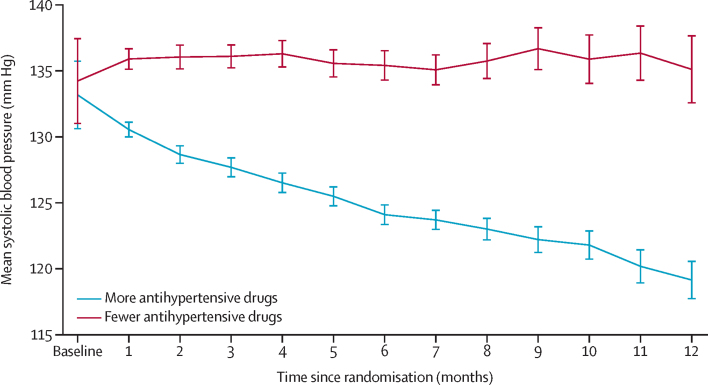
Mean systolic blood pressure in the two treatment groups The trajectory was plotted with the linear mixed-effects model. The model included random intercept and slope of time. The fixed effects included treatment and interaction of treatment and cubic effect of time. The covariance structure of the model was unstructured. Vertical error bars indicate 95% CIs.

**Figure 3:**
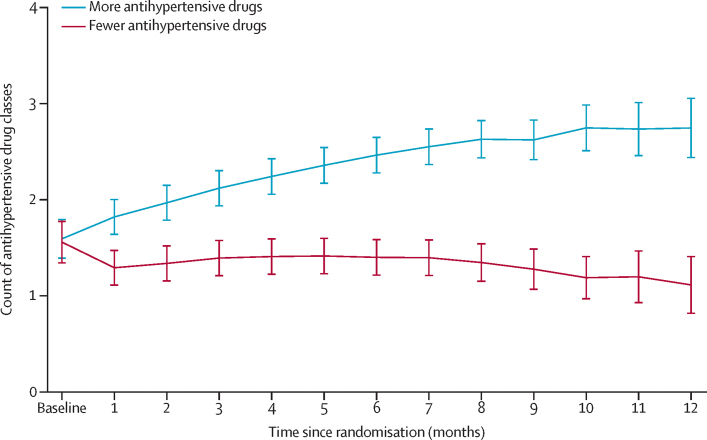
Count of antihypertensive drug classes in the two treatment groups The trajectory was plotted with the linear mixed-effects model. The model included a random intercept and slope of time. The fixed effects included treatment and interaction of treatment and cubic effect of time. The covariance structure of the model was unstructured. Vertical error bars indicate 95% CIs.

Mean systolic blood pressure was gradually reduced in the group assigned to receive more antihypertensive medications and remained largely unchanged in the group assigned to receive fewer antihypertensive medications (figure 2). Mean systolic blood pressure decreased from 134·5 mm Hg (SD 10·7) at baseline to 122·1 mm Hg (10·5) in the group allocated to receive more antihypertensive medications but remained largely unchanged in the group allocated to receive fewer antihypertensive medications, from 134·8 mm Hg (11·2) at baseline to 132·9 mm Hg (15·3) after a duration of 13 months of follow-up after randomisation (appendix p 6). The mean difference in systolic blood pressure between groups was –10·8 mm Hg (95% CI –14·4 to –7·2) at 8 months and –10·7 mm Hg (–17·5 to –4·0) at the end of the 13-month follow-up period. This outcome was achieved with an increase in the number of antihypertensive drug classes from 1·5 (SD 1·1) to 3·0 (1·4) in the more antihypertensive drugs group, compared with a change from 1·5 (1·1) to 1·9 (1·5) in the fewer antihypertensive drugs group (appendix p 7; figure 3). Stratified analyses according to the three strata were generally in line with the overall findings, but there were too few participants in stratum 3 (no change *vs* reduction of two antihypertensive drugs) to enable reliable comparison (appendix pp 11–14).

Frailty scores, as assessed with PRISMA-7 questionnaires, showed no change in the more antihypertensive drugs group, and the difference in frailty scores in the fewer antihypertensive drugs group was not significant (appendix p 5). Similarly, the T-MoCA questionnaire did not reveal any substantial changes in overall or subscale cognitive function assessment. However, the classification of participants into those with or without cognitive impairment seemed unreliable due to large within-group variations (appendix p 9). Health-related quality of life as assessed by EQ-5D-5L remained stable in both groups throughout the follow-up duration (appendix p 10). Self-reported drug compliance was high, with no substantial change over the follow-up duration in either group (appendix p 4).

During the follow-up of 13 months, one cardiovascular event occurred in the more antihypertensive drugs group, compared with six cardiovascular (fatal and non-fatal) events in the fewer antihypertensive drugs group, of which two were fatal (table 3). Three deaths occurred during the follow-up period, one due to cardiac arrest in the group allocated to more antihypertensive drugs and two in the group allocated to fewer antihypertensive drugs (table 3). 33 participants were admitted to hospital for at least one reason other than cardiovascular events, with no difference between the allocated groups (18 [14%] in the more antihypertensive drugs group *vs* 15 [14%] in the fewer antihypertensive drugs group, p=0·97). A high number of non-serious adverse events was reported by participants in the more antihypertensive drugs group (table 3). Notably, the rate of dizziness and fatigue was higher among those allocated to more antihypertensive drugs than to those allocated to fewer antihypertensive drugs (table 3). Other event categories, including falls, fainting, fracture, and confusion, did not differ between the groups (appendix p 3). Those allocated an angiotensin-converting enzyme (ACE) inhibitor, angiotensin receptor blocker (ARB), or diuretics were followed up with a blood test as per routine clinical recommendations. Analysis showed no worsening renal function or electrolyte abnormalities, and average values were stable during the entire study period (appendix pp 17–20).

**Table 3:** Serious adverse events and clinical outcomes of interest

		**More antihypertensive medications (n=126)**	**Fewer antihypertensive medications (n=104)**
**Serious adverse event**			
Hospital admission			
	Myocardial infarction or acute coronary syndrome	0	1 (1%)
	Stroke or transient ischaemic attack	0	3 (3%)
	Heart failure	0	0
	Coronary revascularisation	0	0
	Other hospital admission	18 (14%)	15 (14%)
Deaths			
	Myocardial infarction or acute coronary syndrome	0	1 (1%)
	Stroke or transient ischaemic attack	0	1 (1%)
	Heart failure	0	0
	Coronary revascularisation	0	0
	Other cause of death	1 (1%)	0
**Non-serious adverse event**			
Falls		20 (16%)	17 (16%)
Fracture		4 (3%)	2 (2%)
Dizziness (feeling unsteady or light-headed)		47 (37%)	19 (18%)
Fainting (collapse, syncope, or brief loss of consciousness)		8 (6%)	4 (4%)
Fatigue		13 (10%)	1 (1%)
Loss of consciousness (longer episode of unconsciousness)		4 (3%)	2 (2%)
Delirium or confusion (feeling disoriented, having difficulty paying attention and remembering and making decisions)		6 (5%)	6 (6%)
Loss of balance		4 (3%)	7 (7%)
Nausea		4 (3%)	1 (1%)
Itching		3 (2%)	0
Flushing		1 (1%)	1 (1%)
Headache		3 (2%)	4 (4%)
Fluid retention or leg swelling		6 (5%)	3 (3%)
Bruising (due to fall or other reasons)		5 (4%)	3 (3%)
Shortness of breath		1 (1%)	2 (2%)
Rash		2 (2%)	1 (1%)
Other		41 (33%)	25 (24%)

Data are n (%).

Overall, 201 (87%) of 230 participants described their experience with the trial as very satisfying, and 221 (96%) would consider participating in a trial based on a similar approach again. The decentralised design of the trial and not needing to attend appointments at GP clinics or hospital sites were rated as very satisfying by 223 (97%) participants. The online registration process was rated as very satisfying by 186 (81%) participants and the information available on the designated participant website was rated as very satisfying by 181 (79%).

## Discussion

The ATEMPT trial showed that a substantial lowering of systolic blood pressure can be achieved by use of an IT system for remote recruitment, trial monitoring, and intervention in a cohort of older patients with multimorbidity and polypharmacy—a growing patient group that has been previously under-represented in trials focusing on antihypertensive treatment.^3^, ^4^ On average, a change of one antihypertensive drug was achieved, which corresponded to an 11 mm Hg systolic blood pressure difference between the treatment groups. There was no evidence to suggest that this relatively short-term intervention had an impact on participants' quality of life, cognitive function, frailty status, or medication adherence. However, there were more reports of dizziness and fatigue among those allocated to more antihypertensive drugs than among those allocated to fewer antihypertensive drugs. Although the recruitment phase fell into the second and third wave of the COVID-19 pandemic in the UK, the pilot trial exceeded the anticipated recruitment rate, with a final number of 230 randomly assigned participants. Remote delivery of drugs to patients' homes was feasible and acceptable by participants.

How to handle blood pressure treatment in older, often frail people with multimorbidity has been subject to much controversy. A systematic review and meta-analysis of non-randomised studies that investigated associations between blood pressure and the risk of mortality in older patients found evidence for interaction by frailty status, suggesting that low blood pressure might be harmful in this patient group.^20^ However, these findings were only hypothesis-generating due to the limitations of the study design. SPRINT and HYVET are two randomised trials that have reported outcomes stratified by frailty status.^21^, ^22^ Although these studies showed no evidence of interaction by categories of frailty, SPRINT has been criticised for its method of measuring blood pressure and HYVET was confined to patients with very high blood pressure at baseline. Individual participant data meta-analyses of large-scale randomised controlled trials have not shown any important treatment interaction by age or predicted cardiovascular risk, as proxies for disease burden and frailty.^23^, ^24^ In the meantime, a few studies^25^, ^26^ have assessed the effect of deprescribing in older patients with multimorbidity. Although these studies have overall concluded that deprescribing is feasible and safe, the findings have not been conclusive for several reasons. First, like our study, they have been too small or too short in duration to detect modest differences in important clinical outcomes. Second, intervention fidelity has been suboptimal. For instance, in the ECSTATIC trial, 67% of participants stopped the study-allocated intervention and only 27% were able to maintain the study-allocated intervention throughout the 2-year follow-up.^25^ Similarly, in OPTIMISE, although initial deprescribing was complete, there was a 44% re-prescription rate over the relatively short 12-week follow-up period.^26^ Unsurprisingly, these studies were not able to detect a meaningful difference in blood pressure and are therefore prone to type 2 statistical error.

By contrast, we aimed for a difference of two drug classes between groups, which led to a significant reduction in systolic blood pressure of about 10 mm Hg. However, this change was largely due to the successful addition of drugs in the more antihypertensive drugs group rather than any deprescribing effect in the other group. Indeed, blood pressure in the fewer antihypertensive drugs group remained largely the same throughout the trial. One reason for this result was that, similar to previous deprescribing trials, patients were less willing to have their long-term antihypertensive treatment stopped than new medications added. In some cases, stopping medication led to participants expressing concern about negative consequences arising from an increase in systolic blood pressure and asked their doctor to restart treatments. Another reason for the minimal contribution of deprescribing to the differences between treatment groups is the fact that the proportion of participants with at least one or two antihypertensive drugs that could potentially be stopped was much lower than those in whom treatment could be intensified. Only 19 (8%) of 230 participants belonged to the stratum of no change versus reduction of two antihypertensive drugs and thus did not contribute substantially to the overall results.

The relatively low number of antihypertensive drugs at baseline is consistent with epidemiological studies of the representative UK patient population. An observational study investigating multimorbidity and temporal blood pressure trajectories in people with hypertension in the UK showed that only 2·7% of such individuals were prescribed three or more antihypertensive medications.^27^ On average, people with hypertension and three comorbidities were prescribed 1·5 (SD 0·9) antihypertensives, which is identical to the 1·5 antihypertensive medications at baseline in the ATEMPT population.

Our results did not show a significant difference in the occurrence of serious adverse events between the treatment groups. Although four cases of myocardial infarction, stroke, and transient ischaemic attack were reported in the group with fewer antihypertensive medications, the low number of events did not allow us to test for a statistical difference between the two treatment groups. Clinical trials with a larger number of participants focusing on major cardiovascular events as outcomes are needed to complement the results of our pilot trial. Participants in the group allocated to receive more antihypertensive medications reported a higher number of non-serious adverse events than the treatment group allocated to receive fewer antihypertensive medications. Under those events, the occurrence of dizziness and fatigue was shown to be significantly different between the treatment groups. In the context of an open-label trial, the discrepancy in the reporting of dizziness and fatigue might be attributed to participants' awareness of the changes in their antihypertensive medications and potential expectations of side-effects. Although the remote assessments during the follow-up period were equally scheduled in both treatment groups, participants in the treatment group allocated to receive more antihypertensive drugs had additional contact with the trial team to evaluate the remote delivery of initiated medications and instructions for increasing the dosage, which might also explain the overall greater number of reports in this group. We tried to minimise the risk of adverse effects by favouring the combination of multiple antihypertensive drugs at a low or moderate dose over the full up-titration of single drugs,^18^ but we acknowledge that this approach might not prevent the occurrence of short-lasting adverse effects. The occurrence of dizziness was, however, not associated with an increased reporting of falls, fractures, or episodes of fainting, and did not translate into a meaningful difference in participants' quality of life, cognitive function, frailty status, and medication adherence. Hence, our findings suggest that it is safe to intensify participants' antihypertensive treatment, even in a home-based environment, to thresholds below the typical guideline recommendations for this group.

Key strengths of ATEMPT are the recruitment of an under-represented patient group and the ability to achieve the anticipated systolic blood pressure difference between the two intervention groups. Overall, the remote design of the trial showed a high satisfaction rate among randomly assigned participants. However, we acknowledge that the study was not sufficiently powered to detect more modest differences in safety outcomes and quality of life. Future studies could adopt the decentralised design of ATEMPT and apply digital endpoints for assessment of physical and cognitive functioning. Such outcomes are more sensitive to change and potentially less intrusive. Future studies could also explore enriching recruitment for very old individuals (ie, those aged >85 years) and those with at least moderate frailty in whom treatment uncertainty is substantial.^24^

The trial also has some limitations. Although the findings show that a substantial change in systolic blood pressure can be achieved in this population, the durability of these changes remains unclear due to the relatively short follow-up period of the trial. Future studies with longer-term follow-ups and larger sample sizes could assess the impact of the intervention on clinical outcomes. A further limitation of the pilot trial is its open-label design. However, several measures were implemented to reduce bias. With regard to the clinical frailty assessment based on the PRISMA-7 questionnaire, a limitation can be seen in the lower specificity for detecting frailty in patients compared to other assessment tools, such as the Clinical Frailty Scale. However, the questionnaire was deemed most feasible for a decentralised home-based trial as participants were able to complete this assessment on their own without the direct input of a clinician. Future studies could adopt alternative ways of measuring physical functioning, including with wearables.

Overall, ATEMPT highlighted that patient-centred trials based on digital technologies can successfully recruit and monitor older patients with multimorbidity and polypharmacy. The remotely delivered intervention resulted in a substantial change in systolic blood pressure in older patients with multimorbidity, and this change did not translate into an increased risk of serious adverse events or measurable effects on participants' quality of life, cognitive function, medication adherence, and frailty status. The results of this trial will provide some reassurance about antihypertensive drug use in this patient group and can inform a larger clinical trial that currently is in the planning stages and will focus on assessing major cardiovascular events, safety, physical functioning, and cognitive function.

## Data sharing

The full dataset underlying this Article cannot be shared publicly due to the privacy of individuals who participated in the study. Aggregate and anonymised data will be shared upon reasonable request via email to the corresponding author.

## Declaration of interests

KR reports grants outside the submitted work from the British Heart Foundation, Horizon Europe AI4HF consortium (R79992/CN001), Novo Nordisk Oxford Big Data Partnership, University of Oxford, and UK Research and Innovation's Global Challenge Research Fund (grant reference: ES/P011055/1). KR has previously received consulting fees from Medtronic CRDN, and honoraria or fees from BMJ Heart, PLoS Medicine, AstraZeneca MEA Region, Medscape, and WebMD Medscape UK. MN is supported by a research fellowship from the British Heart Foundation (grant number FS/IPBSRF/22/27060). JP is supported by the British Heart Foundation, outside of the submitted work. DK receives grants from the National Institute for Health and Care Research (NIHR), British Heart Foundation, EU/EFPIA Innovative Medicines Initiative, UK National Health Service, Cook & Wolstenholme Charitable Trust, European Society of Cardiology, Bayer, Amomed, and Protherics Medicines Development, all outside the submitted work. BG and MMA received patient and public involvement payments in line with NIHR guidelines for public contributors. AP-C reports consulting fees from Leantropy. All other authors declare no competing interests.
