# Supplementary material for: Efficacy of decentralised home-based antihypertensive treatment in older adults with multimorbidity and polypharmacy (ATEMPT): an open-label randomised controlled pilot trial
Source: Lancet Healthy Longev. 2024 Mar;5(3):e172–81. doi: 10.1016/S2666-7568(23)00259-3 (PMC11649844; doi:10.1016/S2666-7568(23)00259-3)
Supplement: Supplementary appendix [file mmc1.pdf]

THE LANCET
Healthy Longevity

Supplementary appendix

This appendix formed part of the original submission and has been peer reviewed. We post it as supplied by the authors.

Supplement to: Majert J, Nazarzadeh M, Ramakrishnan R, et al. Efficacy of decentralised home-based antihypertensive treatment in older adults with multimorbidity and polypharmacy (ATEMPT): an open-label randomised controlled pilot trial. *Lancet Healthy Longev* 2024; published online Feb 8. https://doi.org/10.1016/S2666-7568(23)00259-3.

Supplementary materials

Antihypertensive Treatment Evaluation in Multimorbidity and Polypharmacy Trial (ATEMPT): A decentralised open-label pilot trial

Jeannette Majert [1,2], Milad Nazarzadeh [1,2], Rema Ramakrishnan [3], Zeinab Bidel [1,2], Debbie Hedgecott [1,2], Abel Perez-Crespillo [1], Wendy Turpie [1], Naseem Akhtar [1,2], Moira Allison [1], Shishir Rao [1,2], Bernard Gudgin [4] , Melanie McAuley [4] , Christine A'Court [5], Laurent Billot [6], Dipak Kotecha [7,8], John Potter [9], Kazem Rahimi [1,2 *]

[1] Deep Medicine, Oxford Martin School, University of Oxford, UK
[2] Nuffield Department of Women's and Reproductive Health, Medical Science Division, University of Oxford, UK
[3] National Perinatal Epidemiology Unit, University of Oxford, UK
[4] Patient representatives ATEMPT, Oxford, UK
[5] Nuffield Department of Primary Health Sciences, University of Oxford, UK
[6] The George Institute for Global Health, University of New South Wales, Australia
[7] Institute of Cardiovascular Sciences, University of Birmingham, UK
[8] Department of Cardiology & Health Data Research-UK Midlands, University Hospitals Birmingham NHS Foundation Trust, Birmingham, UK
[9] Department of Medicine, University of East Anglia, UK

*** Corresponding author:** Kazem Rahimi

Deep Medicine, Nuffield Department of Women's and Reproductive Health, University of Oxford, Oxford, United Kingdom; email address: kazem.rahimi@wrh.ox.ac.uk; Tel: +44 (0) 1865 617200; Fax: +44 (0) 1865 617202; Postal code and address: 1st Floor, Hayes House, 75 George Street, Oxford, OX1 2BQ, United Kingdom

Contents

Supplementary Table 1. Details of serious and non-serious adverse events. 3

Supplementary Table 2. Drug compliance at 3-monthly intervals. 4

Supplementary Table 3. PRISMA frailty scale at 3-monthly intervals. 5

Supplementary Table 4. Systolic blood pressure reduction at monthly intervals by treatment groups. 6

Supplementary Table 5. Count of antihypertensive drugs at monthly intervals by treatment groups. 7

Supplementary Table 6: Exact rules used by the software for the management of medication changes. 8

Supplementary Table 7. Cognitive function at 3-monthly intervals for analysed participants in the ATEMPT trial. 9

Supplementary Table 8. Health-related quality of life at the 3-monthly intervals for analysed participants in the ATEMPT trial. 10

Supplementary Figure 1. Mean of systolic blood pressure in the two treatment groups over the course of the trial stratified by the 3 post-randomisation strata. 11

Supplementary Figure 2. Count of antihypertensive drug classes in the two treatment groups over the course of the trial stratified by the 3 post-randomisation strata. 14

Supplementary Figure 3. The trend of lab test measurements in the intervention arm over the course of the trial. 17

Supplementary Table 1. Details of serious and non-serious adverse events.

	Arms	
	More antihypertensives (n=126)	Fewer antihypertensives (n=104)
**Serious adverse event**		
**Hospitalisation**		
MI/ACS	0	0
Stroke/TIA	0	3
Heart failure	0	0
Coronary revascularization	0	0
Mitral valve repair	1	0
Diabetic ulcer	1	0
COVID-19 infection	1	0
Rip fracture	0	1
Knee replacement	4	1
Hip replacement	2	1
Shoulder replacement	1	0
TURP	1	0
T-Cell lymphoma	0	1
Loss of consciousness	1	1
Chest infection	1	0
Pelvis fracture	1	0
Cancer screening	0	1
Hip dislocation	0	2
Suspected MI	1	0
Acute cholecystitis	0	1
Inguinal hernia repair	0	1
Exacerbation cGVHD	0	1
Urinary tract infection	0	1
Umbilical hernia repair	1	0
Seizure	1	0
Vomiting	0	1
Fall	0	1
Pneumonia	1	0
**Deaths**		
MI/ACS	0	1
Stroke/TIA	0	1
Heart failure	0	0
Coronary revascularization	0	0
Cardiac arrest	1	0
**Non-serious adverse event**		
Falls	20 (15.8%)	17 (16.3%)
Fracture	4 (3.1%)	2 (1.9%)
Dizziness	47 (37.3%)	20 (19.2%)
Fainting	8 (6.3%)	2 (1.9%)
Loss of consciousness	4 (3.1%)	2 (1.9%)
Collapse	3 (2.3%)	3 (2.8%)
Delirium/confusion	6 (4.7%)	6 (5.7%)
Headache/flushing	2 (1.6%)	4 (3.8%)
Ankle/leg swelling	6 (4.7%)	3 (2.8%)
Joint pain	2 (1.5%)	2 (1.9%)
Dry cough	1 (0.7%)	0
Bruising	1 (0.7%)	1 (0.9%)
Chest pain	1 (0.7%)	1 (0.9%)
Angioedema	1 (0.7%)	0
Atrial fibrillation episodes	3 (2.3%)	1 (0.9%)
Tiredness	5 (3.9%)	1 (0.9%)
Hyperglycaemic episodes	2 (1.5%)	0
Chest infection	3 (2.3%)	1 (0.9%)
Unsteadiness/ imbalance (not related to dizziness)	2 (1.5%)	3 (2.8%)
Erectile dysfunction	1 (0.7%)	0
Eyesight deterioration	0	1 (0.9%)
Shortness of breath	2 (1.5%)	2 (1.9%)
Acid reflux	1 (0.7%)	0
Paraesthesia	1 (0.7%)	0
Covid-19 infection associated short-term memory function problem	1 (0.7%)	0
Rash	3 (2.3%)	1 (0.9%)
Reduced mobility	0	1 (0.9%)
Migraine	1 (0.7%)	0
Urinary frequency	1 (0.7%)	0
Lack of energy	4 (3.1%)	0
Urinary tract infection	2 (1.5%)	3 (2.8%)
Flatulence/constipation	1 (0.7%)	0
Gynaecomastia	1 (0.7%)	0
Short-term visual disturbance	1 (0.7%)	0
Nausea	2 (1.5%)	0
Cold hands	1 (0.7%)	0
Blepharitis	1 (0.7%)	0
Itching	2 (1.5%)	0
Gout episode	2 (1.5%)	0
Skin discoloration	1 (0.7%)	0
Diarrhoea	3 (2.3%)	0
Raynaud's syndrome episode	1 (0.7%)	0
Asthma exacerbation	0	1 (0.9%)
Mood swings	1 (0.7%)	0
Urticaria	1 (0.7%)	0
Night sweats	0	1 (0.9%)

n: number of participants; MI: myocardial infarction; ACS: acute coronary syndrome; TIA: transient ischaemic attack; TURP: transurethral resection of the prostate

Supplementary Table 2. Drug compliance at 3-monthly intervals.

	More antihypertensives		Fewer antihypertensives	
**Time intervals**	Mean [SD]/ median [IQR]	n	Mean [SD]/ median [IQR]	n
Baseline	4.2 (1.5)/5 (0)	126	4.1 (1.6)/5(0)	106
Month 6	4.6 (0.9)/5 (0)	85	4.2 (1.5)/5(0)	59
Month 9	4.6 (1.0)/5(0)	107	4.0 (1.6)/5(2)	93
Month 12	4.8 (0.85)/5(0)	44	4.0 (1.7)/5(3)	30

Responses to the Likert scale adherence question converted to a score for each treatment arm.

n: number of participants; SD: standard deviation; IQR: interquartile range

Supplementary Table 3. PRISMA frailty scale at 3-monthly intervals.

	**More antihypertensives**		**Fewer antihypertensives**	
**Time intervals**	Mean [SD]/ median [IQR]	**n**	Mean [SD]/ median [IQR]	**n**
Baseline	2.6 (1.4)/2 (2)	126	2.8 (1.7)/2(2)	104
Month 6	2.9 (1.6)/2.5 (2.5)	71	3.2 (1.8)/3.5(3.5)	52
Month 9	2.8 (1.4)/2(2)	85	3.0 (1.8)/2(2)	71
Month 12	2.6 (1.5)/2(2)	30	3.1 (2.0)/2(3.1)	24

n: number of participants; SD: standard deviation; IQR: interquartile range

Supplementary Table 4. Systolic blood pressure reduction at monthly intervals by treatment groups.

	More antihypertensives		Fewer antihypertensives		Mean difference (95% CI)	P-value
	Mean (SD)	n	Mean (SD)	n		
Baseline	133.1 (14.4)	126	134.2(16.5)	104	- 1.0 (-5.0 to 2.9)	0.60
1-30	131.8 (11.4)	123	134.8 (12.9)	104	- 3.0 (-6.2 to 0.1)	0.06
31-60	127.7 (12.5)	118	136.2 (11.8)	99	- 8.4 (-11.7 to -5.1)	<0.001
61-90	125.8 (11.6)	113	136.9 (12.8)	96	-11.0 (-14.3 to -7.7)	<0.001
91-120	125.6 (11.7)	116	136.8 (15.3)	97	-11.1 (-14.8 to -7.5)	<0.001
121-150	124.2 (11.2)	112	137.8 (14.4)	92	-12.1 (-15.9 to -8.5)	<0.001
151-180	124.2 (11.2)	112	137.8 (14.4)	89	-13.5 (-17.1 to -9.9)	<0.001
181-210	124.1 (10.8)	102	135.0 (14.5)	82	-10.9 (-14.6 to -7.2)	<0.001
211-240	123.2 (10.2)	88	134.0 (12.9)	74	-10.8 (-14.4 to -7.2)	<0.001
241-270	123.8 (11.2)	75	134.0 (14.6)	61	-10.2 (-14.6 to -5.8)	<0.001
271-300	124.9 (11.1)	55	132.0 (12.6)	42	-7.1 (-11.9 to -2.3)	0.004
301-330	121.0 (10.7)	44	132.7 (14.8)	37	-11.6 (-17.3 to -5.9)	<0.001
331-end of follow-up	122.1 (10.5)	32	132.9 (15.3)	28	-10.7 (-17.5 to -4.0)	0.002

Mean: The average of all repeated systolic blood pressure measurements taken during each time interval.

P-value: Estimated using independent sample t-test

SD: standard deviation; n: number; CI: 95% confidence interval

Supplementary Table 5. Count of antihypertensive drugs at monthly intervals by treatment groups.

	**More antihypertensives**			**Fewer antihypertensives**			
	Mean (SD)	Median (IQR)	n	Mean (SD)	Median (IQR)	n	P-value
Baseline	1.5 (1.1)	1 (1– 2)	126	1.5 (1.1)	1 (1– 2)	104	0.92
1-30	2.3 (1.1)	2 (1– 3)	123	1.5 (1.2)	1 (1– 2)	102	<0.001
31-60	2.4 (1.1)	2 (2– 3)	122	1.6 (1.1)	1 (1– 2)	100	<0.001
61-90	2.6 (1.2)	2 (2– 3)	122	1.6 (1.2)	1 (1– 2)	99	<0.001
91-120	2.7 (1.2)	3 (2– 4)	121	1.6 (1.2)	1 (1– 2)	98	<0.001
121-150	2.8 (1.2)	3 (2– 4)	121	1.6 (1.3)	1 (1– 2)	97	<0.001
151-180	2.7 (1.2)	3 (2– 4)	118	1.7 (1.3)	1 (1– 3)	95	<0.001
181-210	2.8 (1.4)	3 (2– 4)	110	1.6 (1.3)	1 (1– 2)	87	<0.001
211-240	2.9 (1.4)	3 (2– 4)	91	1.7 (1.3)	2 (1– 2)	77	<0.001
241-270	3.0 (1.3)	3 (2– 4)	67	1.8 (1.4)	2 (1– 3)	61	<0.001
271-300	3.0 (1.4)	3 (2– 4)	54	1.9 (1.4)	2 (1– 3)	49	<0.001
301-end of trial	3.0 (1.4)	3 (2– 4)	47	1.9 (1.5)	2 (1– 3)	40	0.001

SD: standard deviation, n: number, IQR: interquartile range

Supplementary Table 6: Exact rules used by the software for the management of medication changes.

Estimated clinic SBP (mmHg)*	Randomisation stratum	Number of BP drugs (not for any other essential indications)	Target change in number of BP drugs		Final number of BP drugs		Difference in number of BP drugs
			Treatment Arm A	Treatment Arm B	Treatment Arm A	Treatment Arm B	
115-124	3	2	0	-2	2	0	2
115-124	3	3	0	-2	3	1	2
115-124	3	4	0	-2	4	2	2
115-124	2	1	1	-1	2	0	2
125-134	3	3	0	-2	3	1	2
125-134	3	4	0	-2	4	2	2
125-134	1	0	2	0	2	0	2
125-134	2	1	1	-1	2	0	2
125-134	2	2	1	-1	3	1	2
135-144	3	3	0	-2	3	1	2
135-144	3	4	0	-2	4	2	2
135-144	1	0	2	0	2	0	2
135-144	1	1	2	0	3	1	2
135-144	2	2	1	-1	3	1	2
145-154	3	4	0	-2	4	2	2
145-154	1	0	2	0	2	0	2
145-154	1	1	2	0	3	1	2
145-154	2	2	1	-1	3	1	2
145-154	2	3	1	-1	4	2	2
155-164	1	0	2	0	2	0	2
155-164	1	1	2	0	3	1	2
155-164	1	2	2	0	4	2	2

*Calculated by adding 5mmHg to the average of all home monitored SBP during run-in. BP: blood pressure; SBP: systolic blood pressure

Supplementary Table 7. Cognitive function at 3-monthly intervals for analysed participants in the ATEMPT trial.

	**More antihypertensives (n= 126)**					**Fewer antihypertensives (n= 103)**				
	Mean [SD]/ median [IQR]					Mean [SD]/ median [IQR]				
	Baseline	Month 3	Month 6	Month 9	Month 12	Baseline	Month 3	Month 6	Month 9	Month 12
Overall T-MoCA score	19.7(1.8)/20(2)	19.5(1.7)/20(3)	19.8(2.0)/20(2.3)	20.1(1.9)/20(3)	20.8(1.4)/21(2)	19.3(2.2)/20(3)	18.9(3.8)/20(2.5)	19.4(2.2)/20(2.8)	20.3(1.9)/21(3)	19.5(3.0)/20.7(3.1)
Subscales:										
Digit span	1.9(0.2)/2(0)	2(0)/2(0)	1.9(0.2)/2(0)	1.9(0.2)/2(0)	1.9(0.1)/2(0)	1.9(0.2)/2(0)	1.9(0.2)/2(0)	1.9(0.2)/2(0)	1.9(0.3)/2(0)	1.8(0.4)/2(0)
Attention	5.3(0.9)/6(1)	5.5 (0.7)/6(1)	5.3(0.8)/6(1)	5.4(0.6)/6(1)	5.6(0.6)/6(0.6)	5.2(0.9)/6(1)	5.4(1.1)/6(0.5)	5.4(0.8)/6(1)	5.4(0.8)/6(1)	5.2(1.2)/6(1)
Repetition	1.5(0.5)/2(1)	1.6(0.5)/2(1)	1.7(0.4)/2(1)	1.7(0.4)/2(0.3)	1.8(0.4)/2(0)	1.5(0.5)/2(1)	1.6(0.5)/2(1)	1.5(0.6)/2(1)	1.7(0.5)/2(0.5)	1.6(0.5)/2(0.8)
Verbal fluency	0.7(0.4)/1(0)	0.8(0.3)/1(0)	0.7(0.4)/1(0)	0.8(0.3)/1(0)	0.8(0.3)/1(0)	0.7(0.4)/1(1)	0.7(0.4)/1(0.5)	0.6(0.4)/1(1)	0.7(0.4)/1(0.5)	0.6(0.4)/1(1)
Abstraction	1.9(0.2)/2(0)	1.8(0.3)/2(0)	1.9 (0.2)/2(0)	1.9 (0.2)/2(0)	1.9(0.2)/2(0)	1.8(0.3)/2(0)	1.7(0.6)/2(0)	1.8 (0.3)/2(0)	1.9 (0.2)/2(0)	1.8(0.3)/2(0)
Recall	4.1(1.1)/5(1)	4.1(0.9)/4(2)	4.0 (1.3)/4.7(2)	4.2(1.1)/5(1)	4.5(0.7)/5(1)	3.9(1.4)/5(2)	3.7(1.7)/4(2)	3.9(1.4)/4(1.5)	4.3(1.1)/5(1)	4.3(0.9)/5(1)
Orientation	5.7(0.5)/6(0)	5.3(0.8)/6(1)	5.7 (0.4)/6(0.5)	5.7 (0.4)/6(0.5)	5.7(0.5)/6(0.1)	5.5(0.6)/6(1)	5.2(1.1)/6(1)	5.6(0.5)/6(1)	5.7(0.4)/6(0)	5.4(0.8)/6(1)

SD: standard deviation; IQR: interquartile range

Supplementary Table 8. Health-related quality of life at the 3-monthly intervals for analysed participants in the ATEMPT trial.

	**More antihypertensives (n=125)**					**Fewer antihypertensives (n=104)**				
	Mean [SD]/ median [IQR]					Mean [SD]/ median [IQR]				
	Baseline	Month 3	Month 6	Month 9	Month 12	Baseline	Month 3	Month 6	Month 9	Month 12
EQ-5D-5L										
Heath state index score	0.7(0.1)/0.7(0.1)	0.8(0.1)/0.8(0.1)	0.7(0.1)/0.7(0.1)	0.7(0.1)/0.7(0.1)	0.7(0.1)/0.7(0.1)	0.7(0.1)/0.7(0.1)	0.8(0.1)/0.8(0.1)	0.7(0.1)/0.7(0.2)	0.7(0.1)/0.7(0.1)	0.7(0.1)/0.7(0.2)
VAS score	78.1(13.2)/80(17)	82.8(8.5)/80.5(9)	76.3(13.7)/80(16.2)	78.3(13.5)/80(20)	73.1(15.0)/75(17.5)	77.9(14.7)/80(20)	84.6(13.4)/89.5(4.7)	73.7(15.6)/76(19.7)	76.5(16.7)/80(17.5)	72.3(21.2)/79.2(26.2)
Subscale:										
Mobility	1.6(0.9)/1(1)	1.5(0.7)/1(1)	1.7(0.9)/1.9(1)	1.6(0.8)/1(1)	2.0(1.0)/2(1.7)	1.8(0.8)/2(1.2)	1.4(0.5)/1(1)	1.9(0.8)/2(2)	1.8(0.9)/2(1)	2.0(0.9)/2(2)
Self-care	1.1(0.4)/1(0)	1.2(0.4)/1(0)	1.2(0.5)/1(0)	1.1(0.5)/1(0)	1.2(0.4)/1(0)	1.1(0.4)/1(0)	1.1(0.3)/1(0)	1.2(0.4)/1(0)	1.2(0.5)/1(0)	1.3(0.6)/1(0.6)
Usual activities	1.5(0.7)/1(1)	1.5(0.5)/1.5(1)	1.6(0.7)/1(1)	1.6(0.7)/1(1)	1.9(1.0)/2(2)	1.6(0.8)/1(1)	1.5(0.6)/1.5(1)	1.8(0.8)/2(1)	1.6(0.8)/1(1)	1.9(0.9)/2(1.6)
Pain/discomfort	1.9(0.8)/2(1)	1.7(0.8)/1.5(1)	2.0(0.7)/2(0)	1.9(0.7)/2(1)	2.3(0.7)/2(1)	2.0(0.8)/2(2)	1.7(0.8)/1.7(1.2)	2.1(0.7)/2(1)	1.9(0.8)/2(1)	2.0(0.7)/2(1.2)
Anxiety/ depression	1.3(0.6)/1(1)	1.2(0.4)/1(0)	1.2(0.5)/1(0.1)	1.3(0.5)/1(1)	1.4(0.6)/1(1)	1.3(0.5)/1(1)	1.3(0.8)/1(0)	1.6(0.7)/1.5(1)	1.3(0.5)/1(1)	1.5(0.7)/1(1)

SD: standard deviation; IQR: interquartile range

Supplementary Figure 1. Mean of systolic blood pressure in the two treatment groups over the course of the trial stratified by the 3 post-randomisation strata.

Stratum 1: No change versus increases of two antihypertensive drug classes

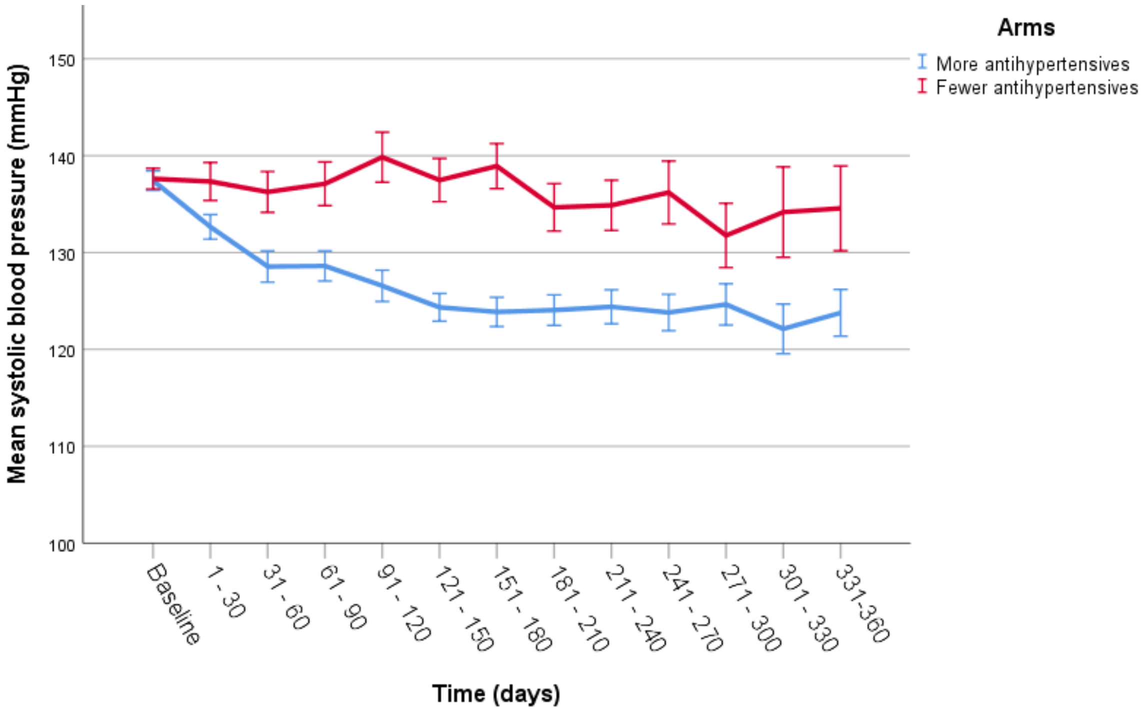

		Arms					
		More antihypertensives			Fewer antihypertensives		
		Systolic blood pressure			Systolic blood pressure		
		Number of participants	Mean	Standard Deviation	Number of participants	Mean	Standard Deviation
Time (Days)	Before- Baseline	80	137	16	56	138	15
	1 - 30	78	133	14	56	137	16
	31 - 60	75	129	16	53	136	16
	61 - 90	74	129	14	52	137	17
	91 - 120	77	127	15	53	140	19
	121 - 150	73	124	12	51	137	16
	151 - 180	74	124	13	50	139	16
	181 - 210	65	124	13	46	135	16
	211 - 240	58	124	13	42	135	16
	241 - 270	49	124	12	34	136	17
	271 - 300	36	125	12	21	132	14
	301 - 330	28	122	13	19	134	19
	331- 360	23	124	13	15	135	19

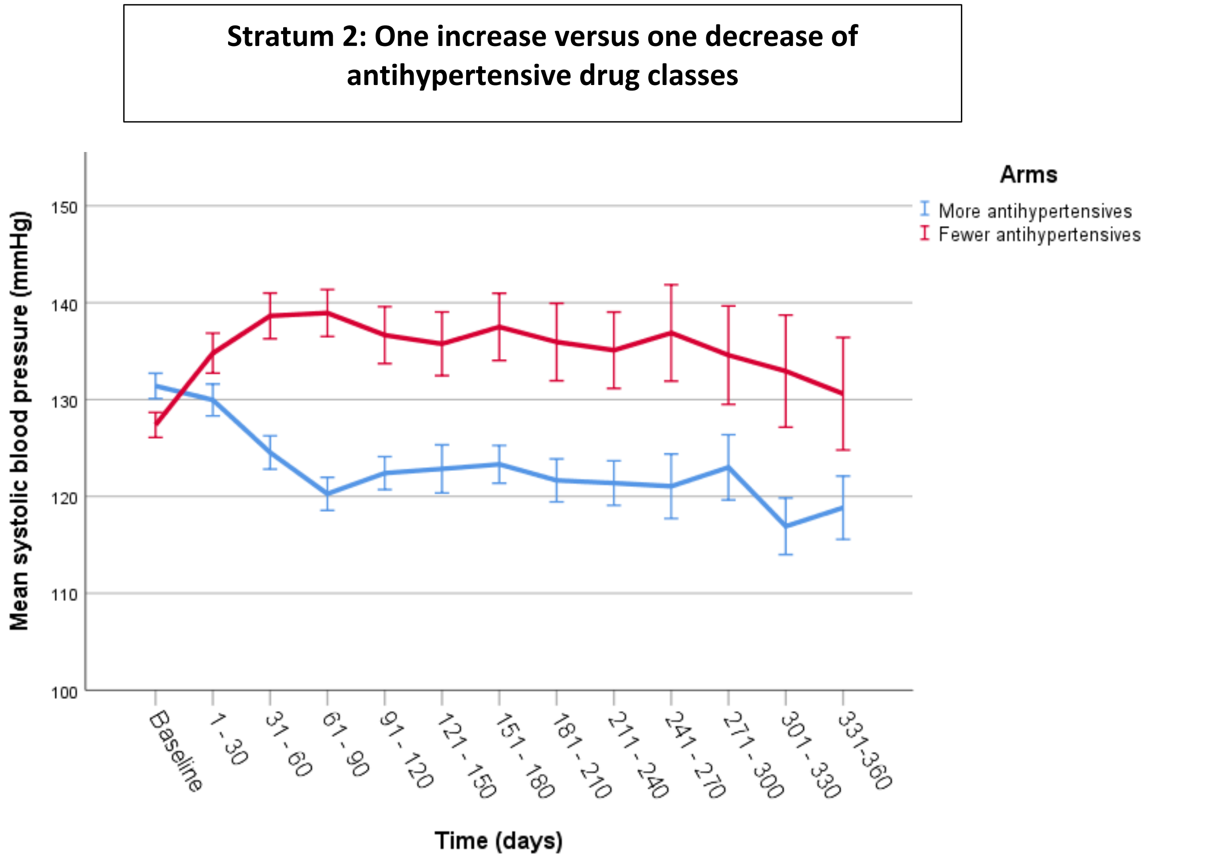

		Arms					
		More antihypertensives			Fewer antihypertensives		
		Systolic blood pressure			Systolic blood pressure		
		Number of participants	Mean	Standard Deviation	Number of participants	Mean	Standard Deviation
Time (Days)	Before-Baseline	37	131	14	38	127	14
	1 - 30	36	130	13	38	135	16
	31 - 60	34	125	12	36	139	16
	61 - 90	31	120	12	35	139	17
	91 - 120	31	122	11	35	137	18
	121 - 150	31	123	16	32	136	18
	151 - 180	30	123	12	31	138	19
	181 - 210	29	122	13	29	136	21
	211 - 240	24	121	13	26	135	18
	241 - 270	20	121	15	21	137	20
	271 - 300	15	123	14	16	135	19
	301 - 330	13	117	11	14	133	19
	331-360	10	119	11	12	131	19

Stratum 3: No change versus decreases of two antihypertensive drug classes

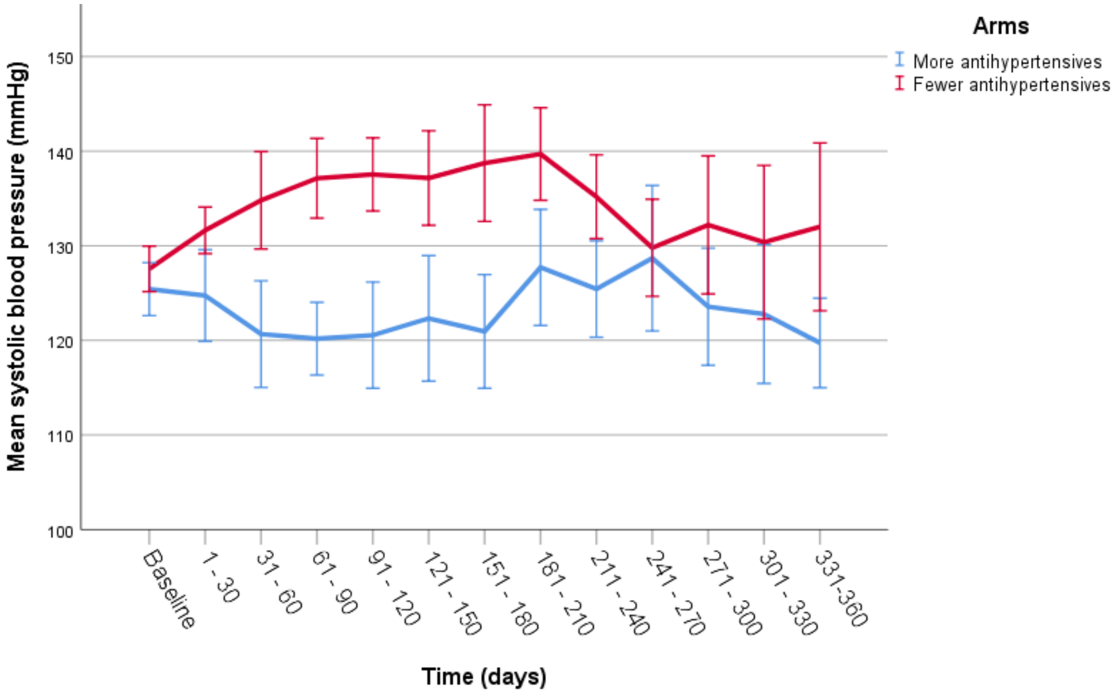

		Arms					
		More antihypertensives			Fewer antihypertensives		
		Systolic blood pressure			Systolic blood pressure		
		Number of participants	Mean	Standard Deviation	Number of participants	Mean	Standard Deviation
Time (days)	Before-Baseline	9	125	14	10	128	14
	1 - 30	9	125	15	10	132	12
	31 - 60	9	121	16	10	135	18
	61 - 90	8	120	12	9	137	16
	91 - 120	8	121	16	9	138	16
	121 - 150	8	122	19	9	137	19
	151 - 180	8	121	18	8	139	20
	181 - 210	8	128	17	7	140	16
	211 - 240	6	125	13	6	135	11
	241 - 270	6	129	16	6	130	12
	271 - 300	4	124	12	5	132	16
	301 - 330	3	123	10	4	130	15
	331- 360	2	120	7	4	132	18

Supplementary Figure 2. Count of antihypertensive drug classes in the two treatment groups over the course of the trial stratified by the 3 post-randomisation strata.

Stratum 1: No change versus increases of two antihypertensive drug classes

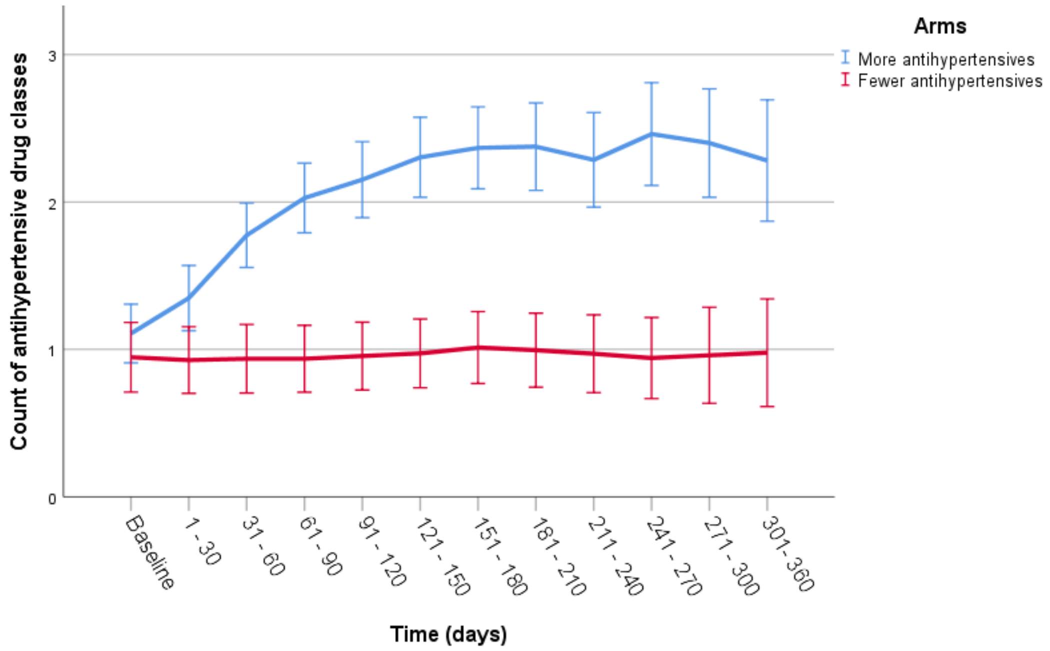

		Arms									
		More antihypertensives					Fewer antihypertensives				
		Count of antihypertensive drug classes					Count of antihypertensive drug classes				
		Number of participants	Mean	Standard Deviation	Median	Range	Number of participants	Mean	Standard Deviation	Median	Range
Time (days)	Before- Baseline	79	1	1	1	3	56	1	1	1	4
	1 - 30	79	1	1	1	4	55	1	1	1	3
	31 - 60	79	2	1	2	4	55	1	1	1	4
	61 - 90	79	2	1	2	5	55	1	1	1	4
	91 - 120	77	2	1	2	5	55	1	1	1	4
	121 - 150	78	2	1	2	5	55	1	1	1	4
	151 - 180	78	2	1	2	5	54	1	1	1	4
	181 - 210	71	2	1	2	5	52	1	1	1	4
	211 - 240	67	2	1	2	5	44	1	1	1	4
	241 - 270	51	2	1	3	5	39	1	1	1	4
	271 - 300	40	2	1	3	5	25	1	1	1	3
	301-360	32	2	1	2	5	22	1	1	1	3

Stratum 2: One increase versus one decrease of antihypertensive drug classes

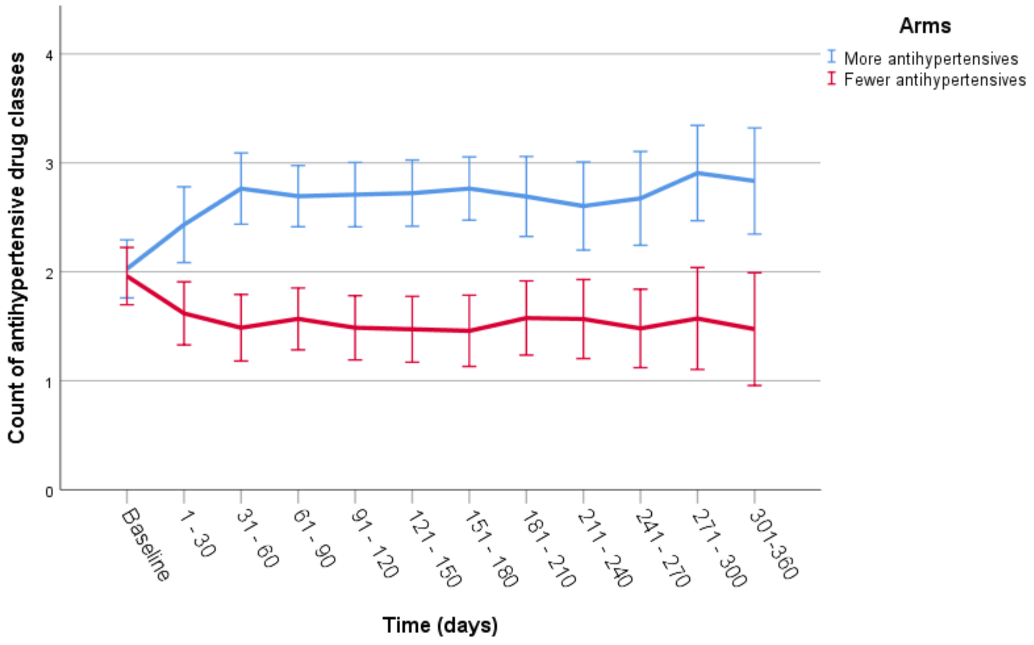

		Arms									
		More antihypertensives					Fewer antihypertensives				
		Count of antihypertensive drug classes					Count of antihypertensive drug classes				
		Number of participants	Mean	Standard Deviation	Median	Range	Number of participants	Mean	Standard Deviation	Median	Range
Time (days)	Before- Baseline	37	2	1	2	3	38	2	1	2	3
	1 - 30	37	2	1	2	3	38	2	1	2	3
	31 - 60	36	3	1	3	3	37	1	1	1	3
	61 - 90	36	3	1	3	3	37	2	1	1	3
	91 - 120	36	3	1	3	4	37	1	1	1	3
	121 - 150	36	3	1	3	4	36	1	1	1	3
	151 - 180	36	3	1	3	4	36	1	1	1	4
	181 - 210	34	3	1	3	4	33	2	1	1	4
	211 - 240	29	3	1	3	4	30	2	1	1	4
	241 - 270	23	3	1	3	4	25	1	1	1	3
	271 - 300	16	3	1	3	2	21	2	1	1	4
	301- 360	15	3	1	3	2	19	1	1	1	4

Stratum 3: No change versus decreases of two antihypertensive drug classes

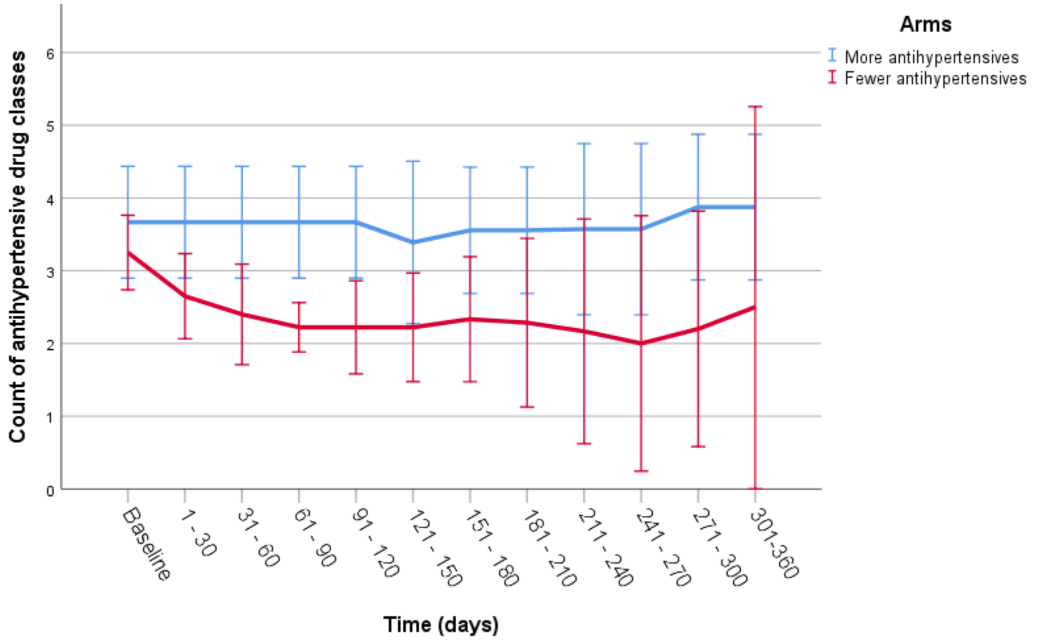

		Arms									
		More antihypertensives					Fewer antihypertensives				
		Count of antihypertensive drug classes					Count of antihypertensive drug classes				
		Number of participants	Mean	Standard Deviation	Median	Range	Number of participants	Mean	Standard Deviation	Median	Range
Time (days)	Before-Baseline	9	4	1	4	3	10	3	1	3	3
	1 - 30	9	4	1	4	3	10	3	1	2	2
	31 - 60	9	4	1	4	3	10	2	1	3	3
	61 - 90	9	4	1	4	3	9	2	0	2	1
	91 - 120	9	4	1	4	3	9	2	1	2	3
	121 - 150	9	3	1	4	5	9	2	1	2	3
	151 - 180	9	4	1	4	3	9	2	1	2	3
	181 - 210	9	4	1	4	3	7	2	1	2	3
	211 - 240	7	4	1	4	3	6	2	1	2	3
	241 - 270	7	4	1	4	3	6	2	2	2	4
	271 - 300	4	4	1	4	2	5	2	1	2	3
	301-360	4	4	1	4	2	4	3	2	3	3

Supplementary Figure 3. The trend of lab test measurements in the intervention arm over the course of the trial.

Serum potassium (mmol/l)

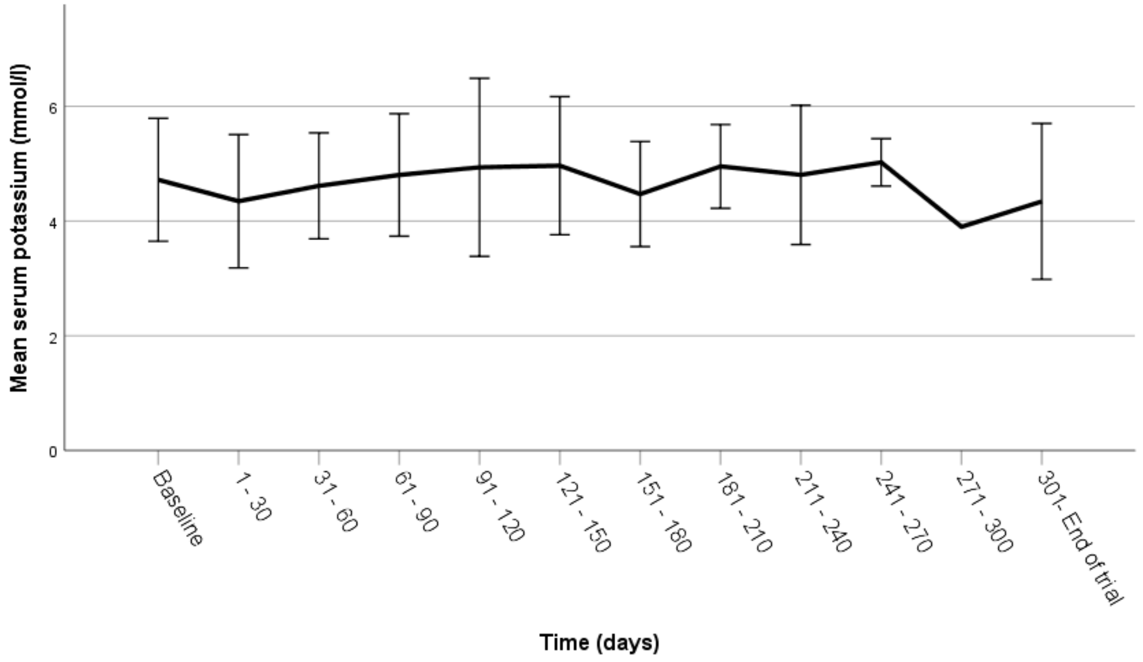

		Mean serum potassium (mmol/l)		
		Number of participants	Mean	Standard Deviation
Time (days)	Baseline	122	4.72	0.54
	1 - 30	12	4.35	0.58
	31 - 60	24	4.61	0.46
	61 - 90	25	4.80	0.53
	91 - 120	15	4.94	0.78
	121 - 150	10	4.97	0.60
	151 - 180	12	4.47	0.46
	181 - 210	9	4.95	0.36
	211 - 240	4	4.81	0.61
	241 - 270	3	5.02	0.21
	271 - 300	1	3.90	NA
	301- End of trial	5	4.34	0.68

Serum creatinine (µmol/l)

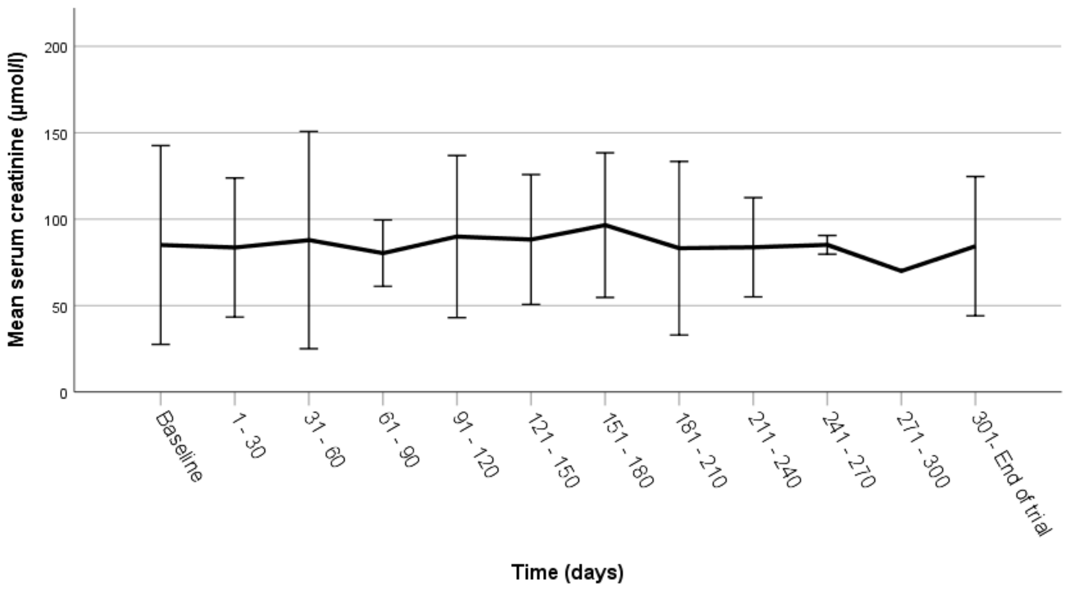

		Mean serum creatinine (µmol/l)		
		Number of participants	Mean	Standard Deviation
Time (days)	Baseline	122	85	29
	1 - 30	12	84	20
	31 - 60	24	88	31
	61 - 90	25	80	10
	91 - 120	15	90	23
	121 - 150	10	88	19
	151 - 180	12	97	21
	181 - 210	9	83	25
	211 - 240	4	84	14
	241 - 270	3	85	3
	271 - 300	1	70	NA
	301- End of trial	5	84	20

Serum urea (mmol/l)

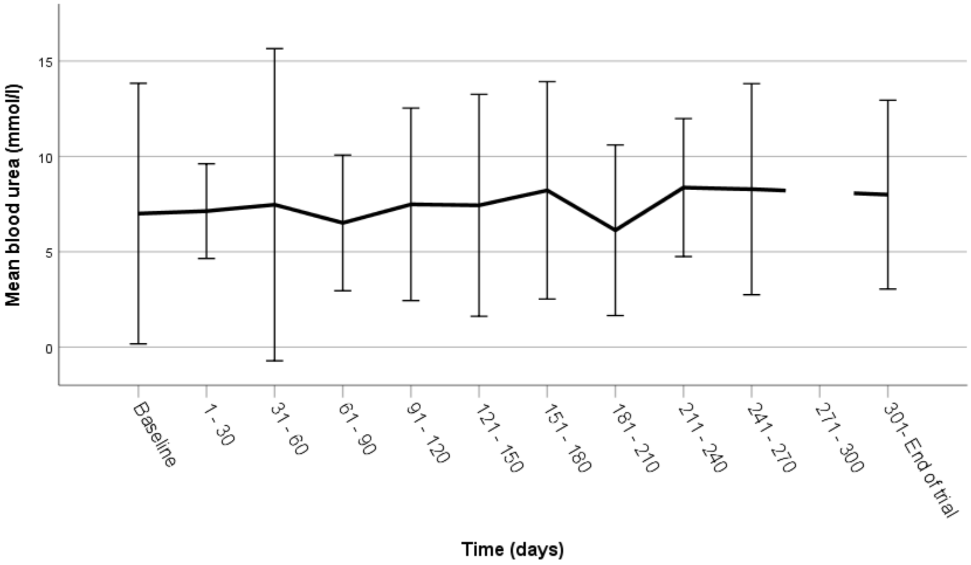

		Mean blood urea (mmol/l)		
		Number of participants	Mean	Standard Deviation
Time (days)	Baseline	122	7	3
	1 - 30	12	7	1
	31 - 60	24	7	4
	61 - 90	25	7	2
	91 - 120	15	7	3
	121 - 150	10	7	3
	151 - 180	12	8	3
	181 - 210	9	6	2
	211 - 240	4	8	2
	241 - 270	3	8	3
	271 - 300	1	NA	NA
	301- End of trial	5	8	2

Serum sodium (mmol/l)

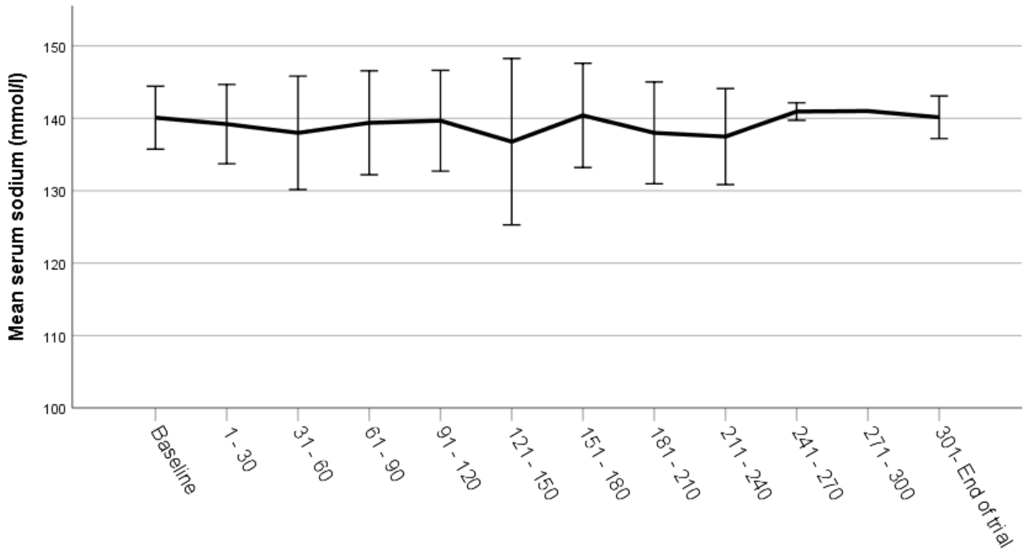

		Mean serum sodium (mmol/l)		
		Number of participants	Mean	Standard Deviation
Time (days)	Baseline	122	140.1	2.2
	1 - 30	12	139.2	2.7
	31 - 60	24	138.0	3.9
	61 - 90	25	139.4	3.6
	91 - 120	15	139.7	3.5
	121 - 150	10	136.8	5.7
	151 - 180	12	140.4	3.6
	181 - 210	9	138.0	3.5
	211 - 240	4	137.5	3.3
	241 - 270	3	140.9	0.6
	271 - 300	1	141.0	NA
	301- End of trial	5	140.1	1.5

Trial Title: Antihypertensive Treatment in Elderly Multimorbid Patients: a pilot study

Trial Acronym: **ATEMPT**: **A**ntihypertensive **T**reatment in **E**lderly **M**ultimorbid **P**atients **T**rial

Ethics Ref: 20/NW/0344
IRAS Project ID: 284172
Clinical Trial Registration Number: ISRCTN17647940
Date and Version No: 26 April 2021 Version 3.0

Chief Investigator: Kazem Rahimi
Nuffield Department of Women's & Reproductive Health
University of Oxford
Level 3 Women's Centre, John Radcliffe Hospital
Oxford OX3 9DU United Kingdom
kazem.rahimi@wrh.ox.ac.uk
Tel: +44 (0) 1865 610297

Investigator: Kazem Rahimi, University of Oxford, UK

Sponsor: University of Oxford
Clinical Trials and Research Governance (CTRG)
Joint Research Office
1st floor, Boundary Brook House
Churchill Drive
Headington
Oxford OX3 7GB
ctrg@admin.ox.ac.uk

Funder: NIHR Oxford BRC, Oxford Martin School

Chief Investigator Signature: 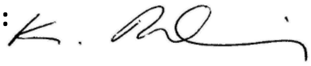

Statistician Signature: 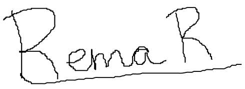

Confidentiality Statement
This document contains confidential information that must not be disclosed to anyone other than the Sponsor, the Investigator Team, HRA, host organisation, and members of the Research Ethics Committee, unless authorised to do so.

TABLE OF CONTENTS

1 KEY CONTACTS 5

2 LAY SUMMARY 6

3 SYNOPSIS 7

4 ABBREVIATIONS 9

5 BACKGROUND AND RATIONALE 10

6 OBJECTIVES AND OUTCOME MEASURES 12

6.1 Aims and objectives 12

6.2 Outcome measures and assessment 13

7 STUDY DESIGN 14

8 PARTICIPANT IDENTIFICATION 14

8.1 Study Participants 14

8.2 Inclusion Criteria 14

8.3 Exclusion Criteria 15

9 PROTOCOL PROCEDURES 15

9.1 Overview 15

9.2 Pre-Screening and Recruitment 15

9.3 Registration and Pre-Eligibility Assessment 16

9.4 Informed Consent 17

9.5 Run-in Phase 17

9.6 Randomisation 18

9.7 Blinding and code-breaking 18

9.8 Description of study interventions and comparators 19

9.9 Communication and delivery of prescription changes, follow-up for BP and safety monitoring......23

9.10 Length of Follow Up Period 24

9.11 Sample Handling 24

9.12 Equipment delivery and set-up 24

9.13 Follow-up for recording of adverse events, patient wellbeing and other study outcomes 24

9.14 Final Visit 25

9.15 Early Discontinuation/Withdrawal of Participants 25

9.16 Definition of End of Study 26

10 SAFETY REPORTING 26

10.1 Definition of Serious Adverse Events 26

10.2 Reporting Procedures for Serious Adverse Events 26

10.3 Non-Serious Adverse Events 26

10.4 Development Safety Update Reports and the Role of DMC........27
11 STATISTICS AND ANALYSIS........27
11.1 Statistical Analysis Plan (SAP)........27
11.2 Description of the Statistical Methods........27
11.3 Sample Size Determination........28
11.4 Analysis populations........29
11.5 The Level of Statistical Significance........29
12 DATA MANAGEMENT........29
12.1 Source Data........29
12.2 Access to Data........29
12.3 The Clinical Trial Management System........29
12.4 Data Processing........30
13 QUALITY ASSURANCE PROCEDURES........31
13.1 Risk assessment........31
13.2 Study monitoring........31
13.3 Study committees........31
14 PROTOCOL DEVIATIONS........32
15 SERIOUS BREACHES........32
16 ETHICAL AND REGULATORY CONSIDERATIONS........32
16.1 Declaration of Helsinki........32
16.2 Guidelines for Good Clinical Practice........32
16.3 Approvals........32
16.4 Other Ethical Considerations........32
16.5 Reporting........33
16.6 Participant Confidentiality........33
16.7 Expenses and Benefits........33
17 FINANCE AND INSURANCE........33
17.1 Funding........33
17.2 Insurance........33
17.3 Contractual arrangements........33
18 PUBLICATION POLICY........33
19 DEVELOPMENT OF A NEW PRODUCT/ PROCESS OR THE GENERATION OF INTELLECTUAL PROPERTY........34
20 ARCHIVING........34
21 REFERENCES........35

22 APPENDIX A: STUDY FLOW CHART 37
23 APPENDIX B: SCHEDULE OF PROCEDURES 38
24 APPENDIX C: AMENDMENT HISTORY 40

1 KEY CONTACTS

**Chief Investigator**	Kazem Rahimi<br>University of Oxford<br>Nuffield Department of Women's & Reproductive Health<br>University of Oxford<br>Level 3 Women's Centre, John Radcliffe Hospital<br>Oxford OX3 9DU, United Kingdom<br>kazem.rahimi@wrh.ox.ac.uk<br>Tel: +44 (0) 1865 610297
**Sponsor**	University of Oxford<br>Clinical Trials and Research Governance (CTRG)<br>Joint Research Office<br>1st floor, Boundary Brook House<br>Churchill Drive<br>Headington<br>Oxford OX3 7GB<br>ctrg@admin.ox.ac.uk
**Funder(s)**	NIHR Oxford BRC, Multimorbidity theme<br>Oxford Martin School, Deep Medicine
**Clinical Trials Unit**	Not applicable
**Trial Statisticians**	Rema Ramakrishnan<br>Zeinab Bidel<br>Milad Nazarzadeh
**Trial Management Group**	Debbie Hedgecott<br>Wendy Turpie<br>Jeannette Majert<br>Naseem Akhtar<br>Jem Chalk<br>Mohammad Manouei<br>Malgorzata Wamil
**Trial Steering Committee**	John Potter, Consultant in Older People's Medicine, Independent Chair<br>Christine A'Court, GP representative<br>Melanie Mcaulay, Patient representative<br>Bernard Gudgin, Patient representative<br>Kazem Rahimi, Chief Investigator<br>Dipak Kotecha , Consultant Cardiologist (DMC Chair)
**Data Monitoring Committee**	Dipak Kotecha: Consultant Cardiologist, Independent Chair<br>Laurent Billot, Independent Statistician<br>John Potter, Consultant in Older People's Medicine (TSC Chair)

2 LAY SUMMARY

Rationale: It is unclear how to best manage blood pressure in older patients or in the presence of many underlying health problems, in particular when blood pressure is not very high.

Aims: This study aims to test whether an intervention that is designed to remotely change participants' medications and deliver them to their homes can lead to important changes in their blood pressure. This information will help to assess whether a larger trial is worthwhile to investigate the effect of the intervention on clinical outcomes. The study additionally aims to assess the acceptability and tolerability of the intervention (using patient-reported outcomes) and to rule out any major excess harms (risk of serious adverse events). Finally, the study aims to test the feasibility and reliability of the remote recruitment, monitoring and follow up procedures without any scheduled clinic visits, and to obtain information about resource requirements for the larger trial.

Setting: This pilot study will take place in the UK with participants from the Thames Valley invited to participate. The study is designed in such way that participants are not required to attend study clinics, and most interactions with study staff are done remotely. Occasional home visits by trained study staff will be scheduled to take a blood sample and to support participants in meeting the study requirements.

Participants: Participants aged 65 years or over with multimorbidity (i.e., three or more underlying health problems) or polypharmacy (5 or more types of medications, excluding any blood pressure medications) willing to have their blood pressure-lowering treatment changed and managed by the trial team. If necessary and desired by participants, their carer will be able to support participants to meet the trial requirements.

Intervention: Participants will be randomised (1:1 ratio) to up to 2 more or 2 fewer blood pressure-lowering medications, taking account of any pre-existing treatment and other indications or contra-indications for blood pressure-lowering treatment. It is expected that the participation lasts between 6 and 12 months.

Study design: A two-armed, parallel group, partially blinded randomised controlled trial

Data collection, analysis and interpretation: Bespoke trial management software will be used for data collection and management. Blood pressure monitoring will be done using a commercially available blood pressure monitor suitable for self-monitoring. The trial team will liaise with participant's doctors and pharmacy services for coordination of medication change and safety monitoring. Data management will be fully compliant with the required confidentiality, security and regulatory requirements. Treatment groups will be compared regarding blood pressure reduction, major clinical outcomes and selected patient-reported outcomes.

Research team: A multidisciplinary research team combining experience from medicine, epidemiology, engineering and clinical trials.

3 SYNOPSIS

Trial Title	Antihypertensive treatment in elderly multimorbid patients: A pilot study
Trial Acronym	ATEMPT
Study registration	ISRCTN17647940
Sponsor	University of Oxford<br>Clinical Trials and Research Governance (CTRG)<br>Joint Research Office<br>1st floor, Boundary Brook House<br>Churchill Drive<br>Headington<br>Oxford OX3 7GB<br>ctrg@admin.ox.ac.uk
Funders	NIHR Oxford BRC, Oxford Martin School
Study Design	A two-armed, parallel group, partially blinded randomised controlled trial. ATEMPT combines a pilot clinical study (blood pressure change and patient-reported outcomes) with a feasibility study (testing of the remote monitoring and follow-up procedures) for a future major clinical outcome trial (assessing study procedures, recruitment, retention and sample size).
Study Participants	Individuals aged 65 years or over with multimorbidity or polypharmacy, a systolic blood pressure (SBP) in the range of 115-165 mmHg at baseline
Intervention(s) and Comparator	Participants will be randomised into two groups of more vs. fewer anti-hypertensive medications. Depending on baseline systolic BP and any pre-existing anti-hypertensive treatments, participants will have the number of their pre-existing anti-hypertensive drug classes reduced, left unchanged or increased, aiming for a difference of two drug classes between groups.
Sample Size	Target of 200 participants
Planned Recruitment period	December 2020 – December 2021

	Objectives	Outcome Measures	Timepoint(s)
Primary objective: Intervention efficacy	To investigate the efficacy of intervention	Modelled difference in mean change in SBP from baseline to end of follow-up between treatment groups, and cumulative time-weighted difference in SBP between treatment groups (**primary outcome**)	Repeated measure (at least weekly)
	To investigate the intensity of treatment	Counts and total daily dose of each anti-hypertensive drug for each individual	Repeated measure (monthly)
Intervention safety and tolerability	To assess safety of intervention	Comparisons of adverse events such as falls and admissions to hospital collected from central	Minimum monthly

		databases and participant questionnaires	
	To assess the acceptably and tolerability of intervention	Completion rates and time taken to completion of electronic questionnaires on health-related quality of life, cognitive function and drug compliance	3-monthly
Identification and recruitment of participants	To assess the efficiency and effectiveness of participant recruitment	Recruitment rate per month<br>Percentage of invitations to participant self-registration, participants screened, participants consenting for run-in, and participants randomised	Monthly until end of recruitment period
Remote monitoring and follow-up procedures	To test the reliability of web-based system to monitor participants remotely	Frequency and range of BP recorded.<br>Proportion of participants requiring home visit or phone calls for technical support	Run-in and end of follow-up
	To investigate the reliability of up-to-date information on drug prescription	Linkage with third party databases and timeliness of information updates and comparison with information gathered from participants	Registration and end of follow-up
Resources	To estimate resources required for the subsequent trial	Sample size<br>Costs and staff time	End of study

4 ABBREVIATIONS

ACC	American College of Cardiology
AHA	American Heart Association
BP	Blood Pressure
CI	Chief Investigator
CRF	Case Report Form
CTIMP	Clinical Trial of an Investigational Medicinal Product
DMC	Data Monitoring Committee
EHR	Electronic Health Records
ESC	European Society of Cardiology
ESH	European Society of Hypertension
GCP	Good Clinical Practice
GDPR	General Data Protection Regulation
GP	General Practitioner
HRA	Health Research Authority
ICD	International Statistical Classification of Diseases and Related Health Problems
IP	Internet Protocol
MHRA	Medicines and Healthcare products Regulatory Agency
NHS	National Health Service
NICE	The National Institute for Health and Care Excellence
PI	Principal Investigator
R&D	NHS Trust R&D Department
RCT	Randomised Controlled Trial
REC	Research Ethics Committee
SAP	Statistical Analysis Plan
SOP	Standard Operating Procedure
TMG	Trial Management Group
TSC	Trial Steering Committee

5 BACKGROUND AND RATIONALE

To date, over 500,000 patients have participated in large-scale randomised trials of blood pressure (BP) lowering treatment.[1] These trials have collectively shown that pharmacological blood pressure reduction is an effective strategy in reducing the risk of cardiovascular events in at-risk populations and that the relative risk reduction afforded by treatment is proportional to the intensity of BP lowering. At the same time, these trials have found no compelling evidence to suggest that the presence or absence of cardiometabolic conditions or participants' BP at baseline modify treatment effects. However, due to restrictions of participant selection in those trials, the importance of BP-lowering treatment in specific patient populations remains uncertain.

One growing population group in whom uncertainty of treatment effects remains high is older patients with multimorbidity, i.e., when several chronic conditions co-occur, in particular when BP is not very high.[2,3] To our knowledge, no trial has investigated BP treatment interaction by number or groups of comorbidities. The only trial that stratified participants by disease counts was the SPRINT trial, but this analysis investigated the effect of intensive treatment on quality of life and adherence (and showed no material difference between groups) but not on cardiovascular or safety outcomes.[4]

In absence of direct evidence on treatment effects in people with multimorbidity, other indirect approaches have been taken by researchers. For instance, multimorbidity partially overlaps with frailty[5] as another complex syndrome of ageing.[6] A systematic review and meta-analysis of non-randomised studies that investigated associations between BP and risk of mortality in older patients found evidence for an interaction by frailty status, suggesting that low BP might be harmful in this patient group.[7] However, these findings were only hypothesis-generating due to limitations of the study design. To date, SPRINT and HYVET remain the only two randomised trials that have reported outcomes by frailty status.[8,9] Although these studies showed no evidence of interaction by categories of frailty, SPRINT has been criticised for its method in measuring BP and HYVET was confined to patients with very high BP at baseline. Evidence for treatment modification by age, as another proxy for disease burden, is also limited. An individual-patient meta-analysis showed no evidence of treatment interaction by age. However, the age range among the trials included was relatively narrow (mean age of all participants 65 years, and 72 years in the group >65 years).[10] Hence, the study had limited power in assessing differential effects at higher age categories.

Patients with multimorbidity are at substantially increased risk of adverse clinical outcomes, including cardiovascular disease.[11] Thus, another indirect way of assessing possible effects of treatment in older multimorbid patients is to stratify patients by their future risk of cardiovascular events, using frailty scores. Such a study was recently reported and found no treatment interaction by baseline risk of cardiovascular disease in an individual-patient data meta-analysis of randomised trials.[12] A limitation of that study, however, was that for those with pre-existing disease (e.g., diabetes mellitus or coronary artery disease), the value of risk-based decision making was not convincing, and overall the average risk of participants for cardiovascular disease was not high. In addition, included trials had limited information about effects on tolerability and safety outcomes, which are common reasons for withholding treatment in elderly and multimorbid patients.[13,14]

Another related open question in older patients with multimorbidity is the optimum BP threshold for treatment initiation or maintenance. In the absence of reliable evidence from RCTs, the debate has been

dominated by conflicting evidence from observational studies.[15-17] Although such studies are often subject to reverse causation and residual confounding, they have contributed to caution in guideline recommendations. The table below summarises the key recommendations from three major clinical practice guidelines for a hypothetical community-dwelling lady who is 80 years old, has a few common comorbidities (e.g., ischaemic heart disease, atrial fibrillation, stage 3 chronic kidney disease, osteoarthritis, treated hypothyroidism and anaemia). Her last clinic blood pressure is 137/72 mmHg while being treated with two anti-hypertensive drugs.

Guideline	Recommendation
ACC/AHA 2017[18]	• Treatment of hypertension with a systolic **BP treatment goal of less than 130 mmHg** is recommended for noninstitutionalized ambulatory community-dwelling adults **(≥65 years of age)** with an average SBP of 130 mmHg or higher<br>• For older adults (≥65 years of age) with hypertension and a **high burden of comorbidity** and limited life expectancy, **clinical judgment, patient preference, and a team-based approach** to assess risk/benefit is reasonable for decisions regarding intensity of BP lowering and choice of antihypertensive drugs
ESC/ESH 2018[19]	Specific recommendation for those >65 years:<br>• When treated, BP should be **lowered to a systolic value of 130–139 mmHg** and a diastolic value of <80 mmHg if tolerated. **Treated SBP values of <130 mmHg should be avoided.**<br>• **In some patients, the best achievable BP may be higher than the recommended target**, but it should be recognised that any amount of BP lowering is likely to be worthwhile and associated with a reduced risk of major CV events (especially stroke and heart failure) and mortality
NICE 2019[20]	• **Consider** starting antihypertensive drug treatment for people aged over 80 with **stage 1 hypertension** (140/90 mmHg to 159/99 mmHg) and maintain that level. Use **clinical judgement** for people with frailty or multimorbidity

As the table shows, these recommendations leave generally much room for judgement. The NICE multimorbidity guidelines go even further and recommend research into withdrawing preventative treatments including antihypertensive drugs as “it is plausible that harms outweigh benefits in some people with multimorbidity (for example, because of higher rates of adverse events in older, frailer people prescribed multiple regular medicines, or because the expected benefit from continuing a preventive medicine is reduced when there is limited life expectancy or high risk of death from other morbidities)”.[21] Several international guidelines and professional societies summarise the evidence and come to the conclusion that more research is needed in the very elderly, multimorbid and frail.[19]

From patients’ perspectives, uncertainty on BP management has major consequences. Would they benefit from initiation or continuation of more drugs for BP treatment in presence of multimorbidity and polypharmacy, or would they be better off with fewer or no anti-hypertensive medicines? What are the consequences of treatment intensification or de-escalation on their quality of life, physical functioning, cognitive functioning and death? From healthcare providers’ point of view, this uncertainty is highly relevant too. In the UK, the clinical management of hypertension accounts for 12% of visits to primary care and up to £2.1 billion of healthcare expenditure, the majority of which is due to BP-lowering drugs, which are one of the most widely prescribed drug groups in the NHS.[22]

One key reason for the existing gap in evidence is the challenge of recruiting sufficiently large numbers of older and multimorbid patients into clinical trials. A recent systematic review that examined phase III clinical trials funded by the National Institutes of Health from 1965 to 2015 concluded that “beyond explicit exclusion by age, older adults were often implicitly excluded based on various comorbid conditions such as polypharmacy/concomitant medication (37%) or cardiac issues (30%)”.[23] Even when trials do not have restrictive inclusion criteria, it is often challenging to recruit older or multimorbid patients into trials. One major barrier to trial participation is the burdensome trial procedures, which often demand regular travel to study clinics. In the context of BP treatment, another barrier is the concern about short-term side effects of treatment, which episodic clinic assessments might not be able to capture. This concern is of particular importance in elderly and multimorbid participants, given their altered drug metabolism, which could lead to exaggerated and fluctuating response to treatment.[24]

Patient-centred trial designs that make use of technological advances could overcome the challenges of participant recruitment, monitoring, and follow-up. For instance, in a previous trial we found that older multimorbid patients with little or no prior experience with digital technologies could be supported to use a tablet computer with a bespoke study application that enabled remote communication with trial staff at any time during the course of the study.[25,26] Often with support from their carers, participants were also able to regularly use a BP monitor and weighing scales that were wirelessly linked to the tablet computer. Such systems have been shown to achieve high acceptability and satisfaction rates among older people with multimorbidity whilst reducing the burden of monitoring on participants and study staff.[25,26] With continued technological advances, monitoring of trial participants in their home environment is becoming even less intrusive and less burdensome. For instance, cuff-less BP devices are being developed to enable monitoring of BP (and a number of other physiological measures) continuously through a wristband. Another emerging solution enables measurement of BP and heart rate by simply asking the participants to place their finger on their smartphone camera for about 30 seconds. Such technologies, once clinically validated, will not only be useful for efficient trial management, but could also enable more dynamic assessment of BP and patient well-being for more tailored treatment recommendations. Indeed, in the context of BP monitoring, evidence has been mounting that within-person variability of BP measurements could have implications for treatment.[27] For instance, even in major landmark RCTs, long-term BP values are substantially different from those used to define treatment thresholds in clinical guidelines,[28] and home-measured values have a stronger predictive ability for cardiovascular outcomes.[29]

This pilot trial will, therefore, test the usability and effectiveness of IT-enabled systems to recruit and monitor participants with little direct physical contact between participants and the study team, and it will also estimate the efficacy, safety and tolerability of intensive BP lowering under remote monitoring conditions. This information will serve to plan an adequately powered major, multinational RCT.

6 OBJECTIVES AND OUTCOME MEASURES

6.1 Aims and objectives

The primary objective of this pilot study is to estimate the effectiveness of the intervention on change in BP and prescribed anti-hypertensive drugs.

Secondary objectives are

1. To assess the acceptability and tolerability of the intervention (using patient-reported outcomes) and to rule out any major excess harms (risk of serious adverse events);
2. To test the feasibility of main components of the trial, namely participant recruitment, randomisation, delivery of treatment, and remote assessment of trial outcomes and to obtain information about resource requirements for the main trial.

6.2 Outcome measures and assessment

The primary outcome measure is home-measured average systolic BP during follow-up. Details of the analysis of the primary outcome will be specified in a statistical analysis plan (see section 11). An overview of all outcome measures for comparison of randomised groups, their definition, and assessment is provided in the table below

Outcomes	Measures & Assessment	Timepoints
Modelled difference in mean change in SBP from baseline to end of follow-up between treatment groups, and cumulative time-weighted difference in SBP between treatment groups (**primary outcome**)	Home-measured BP values	Repeated measurements (at least weekly)
Prescribed anti-hypertensive medication	Counts of each anti-hypertensive drug class for each individual; average daily dose of all anti-hypertensive drugs for each individual.	Repeated measurements (approximately monthly intervals)
Safety assessment (Serious adverse events and selected non-serious adverse events)	All hospitalisations, death, and selected outcomes such as falls, fracture, dizziness and delirium/confusion collected from central databases and participant questionnaires;	Minimum at the end of the study
Treatment acceptability and tolerability	Health-related quality of life, Cognitive function (Modified Telephone Interview for Cognitive Status (T-MoCA), and drug compliance. By default, this information will be collected via the direct completion of the eCRF by participants/carers (with the exception of T-MoCA that is to be applied over the phone or in person). However, where a visit or call to a participant from a member of the study team coincides with a protocol scheduled assessment, information may be collected by study staff and added directly to the eCRF.	3-monthly

In addition to these outcomes intended from comparisons between treatment arms, a range of process outcomes will be collected to assess the feasibility of a planned large-scale trial. These outcomes will be collected by study staff or through analysis of routinely collected information by the trial IT system. Purpose, outcome measure and evaluation are described below.

Outcomes	Measures & Assessment	Timepoints
Identification and recruitment of participants	▪ Recruitment rate per month Percentage of invitations to participant self-registration, participants screened, participants consenting for run-in, and participant randomised	Monthly until end of recruitment period
Remote monitoring and follow-up procedures	▪ Frequency and range of BP recorded,<br>▪ Proportion of participants requiring home visit or phone calls for technical support	Run-in and end of follow-up
	▪ Linkage with national or third party databases and timeliness of information updates and comparison with information gathered from traditional sources	Registration and end of follow-up
Resources (sample size, costs and staff time)	▪ To estimate resources required for the subsequent trial	End of study

7 STUDY DESIGN

ATEMPT is a two-armed, parallel-group, partially blinded randomised controlled trial, comparing more versus fewer anti-hypertensive medications in older patients with multimorbidity or polypharmacy. The trial will have a run-in phase of up to 4-months (to confirm eligibility and to modify the trial IT system if necessary). The trial will be piloted at one site in Oxford, UK. Interactions between participants and study staff will be mainly done remotely with the use of the trial IT system. As necessary, information exchange will be complemented through telephone or home visits.

8 PARTICIPANT IDENTIFICATION

8.1 Study Participants

Participant selection criteria aim to identify a relatively unselected older and multimorbid patient group, for whom there is substantial uncertainty about pharmacological BP treatment. Selection criteria will be assessed in two stages. Initially potential participants will self-assess their suitability to participate via the trial online portal (see section 9.3 Registration and Pre-Eligibility Assessment). If successful and once consent has been provided, participants will be assessed during the run-in phase for adherence to the remaining criteria and to enable the research team to verify the information provided by potential participants during self-assessment (see section 9.5 Run-in Phase).

8.2 Inclusion Criteria

- Age 65 years or more;
- At least three chronic diseases (as specified in section 9.2 Pre-Screening and Recruitment) or at

least 5 non-antihypertensive drugs;
- Baseline office systolic BP in the range of 115-165 mmHg (or home systolic BP of 110-160 mmHg);
- Willingness to have anti-hypertensive medication modified (increased, decreased or left unchanged) by trial team for the duration of the trial;
- Willingness to register with the specified on-line pharmacy service to facilitate remote dispensing of anti-hypertensive medication and period sharing of participants prescription list;
- Willingness and ability to monitor BP at home;
- Participant is willing and able to give informed consent for participation in the trial;
- Participant's or carer's, ability to understand English language is required to operate the IT-enabled (web-based) system.

8.3 Exclusion Criteria

- Previous admission to hospital with primary diagnosis of heart failure or known systolic heart failure;
- Self-reported symptomatic orthostatic hypotension;
- Pre-existing anti-hypertensive treatment, or baseline BP prohibit the achievement of the minimum required change in anti-hypertensive drugs defined as:
  - Office systolic BP 155 to 165 mmHg and on 3 or more anti-hypertensive medications, or
  - Office systolic BP 115 to 124 mmHg and no anti-hypertensive medications;
- Routine use of a dosset box or NOMAD box to help manage medications
- No reliable 4G mobile or WiFi network connectivity at home
- Lack of uncertainty in the opinion of the Investigator for changing BP drug treatment (e.g., any other significant disease, which, in the opinion of the Investigator, may either put the participant at risk because of participation in the trial, or the participant's ability to participate in the trial).
- Current participation in any other research trial that investigates blood pressure management.

9 PROTOCOL PROCEDURES

9.1 Overview

A schematic overview of procedures for participant identification, recruitment and follow-up is provided in APPENDIX B: SCHEDULE OF PROCEDURES .

9.2 Pre-Screening and Recruitment

Potentially eligible participants will be identified via four possible routes. One route will be through the screening of hospital discharge diagnoses by NHS Digital. The study team will provide a search query based on discharge ICD codes, age range and geographic location. More specifically, the search query will select individuals aged 65 years or more with three or more chronic conditions diagnosed within a 5-year time window prior to the latest assessment date. Individuals with a recorded diagnosis of heart failure will be excluded. The selection of chronic conditions of interest will be based on 3-character ICD codes, using the following logic: First, ICD chapters 1 and 15 to 22 will be excluded, which means that only conditions included in chapters 2 to 14 will be considered. Then, individuals who have 3 or more of the conditions recorded on their discharge notes will be selected, considering that each code counts as one disease. The search query will be provided on a number of occasions, each time restricted to individuals who live within different regions in the Thames Valley area and are alive at the time of data sharing with the research team. This will enable the list of potential participants to be provided in smaller, more

manageable batches and permit the contact of all individuals on a given list with a month. Limited identifiable information provided by NHS Digital will be used for further pre-screening. After checking of the data for duplicate records, participants will be invited by post for consideration of participation into the trial.

The second route will involve the on-line pharmacy identifying patients already signed up to receive prescriptions through their service. Patients living in the Thames Valley area who are aged 65 years or more and known to be taking at least 5 non-antihypertensive medications will be approached by the on-line pharmacy and consent will be sought to share contact details with the study team.

The third route will utilise a health research recruitment service who will use targeted criteria to advertise on social media platform(s) in order to highlight the study website to potential participants. Any potential participants who access the website as a result of being made aware of the study via this route will be subject to the same eligibility criteria as invited participants.

A fourth route for participant identification is also possible where participants might directly indicate an interest in study participation by independently accessing the website (e.g., without an invitation letter). Again such participants will be subject to the same eligibility criteria.

Similar to previous studies,[30] GPs in the trial region will be informed about the trial and made aware that some of their patients may be invited to participate. They will be given the option to contact the trial team if they do not wish potentially eligible patients from their practice to be invited or to be more actively involved in the trial management. Postal invitations will be sent out by a specialised mailing company that complies with the data governance standards required by the NHS, the University of Oxford and GDPR. We anticipate that about 2% of invited participants will reach the randomisation stage. Accordingly, invitations will be sent out in batches and a reminder to non-responders within 28 days of initial invitation. The profile of participants recruited in the early stages of the trial might lead to oversampling of the next batch of invitations to focus on underrepresented participant groups (e.g., very elderly, women) in later stages.

9.3 Registration and Pre-Eligibility Assessment

The postal invitation will explain the reason for contacting the participants and invite them to seek further information on the participant online portal. The invitation will make it clear that participants unsure of or unwilling to use the online portal may nominate a friend or carer to assist them with accessing and using the website during the registration process and throughout study participation if required. Additionally, a free phone line and email will be available if participants or their carers would prefer to contact the study team directly for further information. This option will be available at every stage of self-registration and throughout the course of the study.

Where a participant has sought the help of a friend or carer to access the participant portal, they will be able to highlight this during the sign in process.

The invitation will include the URL address of the website to enable access using a PC, tablet or smartphone, as well as a unique access code (UAC). The website will provide general information about the trial and a brief (about 2 minutes) video infographic. If a participant is interested and would like to check their suitability to take part, they will be invited to enter the UAC, which will enable them to

complete an initial registration form and learn more about the study via an interactive participant information sheet including video infographic. The information provided will detail the exact nature of the study; what it will involve for the participant; the implications and constraints of the protocol; any risks involved in taking part. It will be clearly stated that the participant is free to withdraw from the study at any time for any reason without prejudice to future care, without affecting their legal rights, and with no obligation to give the reason for withdrawal.

Participants will have the option of seeking help from study staff (over the phone or in person) at any time during the study. See APPENDIX A: STUDY FLOW CHART for further details regarding the eligibility assessment process.

9.4 Informed Consent

As part of the online registration, participants alone or with assistance from a friend or carer will be required to complete an electronic consent form. The consent form will consist of check boxes to attest that they agree to the different aspects of the trial, and that they have read the online participant information sheet. Within the consent process the participant will be asked to agree to the trial team obtaining access to their health records and to agree to their GP being informed about their participation in the trial. A final check box will read 'I have read, answered and understood all of the above questions and understand this is an electronic signature' and this will need to be checked by the participant before they can continue. The consent form will record the participant's name, date and a time stamp of when the form was electronically submitted. The participant information sheet and signed consent form will be made available in a portable document format (PDF) format to view electronically or print for their own records at the time of completion. Alternatively, at any time during study, the participant can contact the study team to request that a copy is printed and posted to them.

The decision of a participant to participate in clinical research is voluntary and will be based on a clear understanding of what is involved. For the majority of participants, the consent process will be conducted remotely and entirely via the participant portal, without physical contact with study staff. As such, participants will have as much time as they wish to consider their participation, to discuss with family members/carers and to seek further information from the trial team. A free phone line and email address will be available if participants want to contact the study team for further information or clarification.

For some participants, a home visit might be required to complete the trial registration process and to seek consent. During such a visit, a trained study clinician will explain the trial procedures making use of the online system including video infographic and will go on to collect the necessary information to complete the pre-eligibility assessment and seek consent. Home visits will be conducted with reference to guidance provided in our SOP Safety of Research Staff in the Community.

9.5 Run-in Phase

There will be no routine clinic assessments and all monitoring and management of participants by study staff will be done remotely or physically at participants' homes. This will enable inclusion of participants for whom regular attendance to trial clinics is a burden and allow more frequent and efficient collection of trial outcomes directly from participants, which will be necessary for a future main trial to become feasible. In a previous study of multimorbid heart failure patients with a mean age of 76 years we

observed high participant satisfaction with remote monitoring through electronic devices. However, in order to maximise participation satisfaction and participation retention in the trial, participants will be offered different options for follow-up based on their initial responses.

As the default option, participants will be screened for their ability and willingness to use a cuff-based BP monitor. Participants are able to use their own BP monitors provided it is a certified device. Otherwise, a device will be provided to participants for home monitoring. Cuff-based BP monitoring will be considered as gold-standard, but alternative remote monitoring techniques/devices for BP measurement might be considered in some patients if validated for such use or possibly to validate as part of a sub-study.

The run-in phase period will enable the trial team to highlight any issues with the transfer of health data to the trial portal and resolve them before a participant is randomised. During run-in, remaining information for full eligibility assessment will be obtained. In particular, information about previous diagnoses and a complete list of medications will be obtained by nurses during a home visit and later confirmed through linkage with national Electronic Health Records (EHR), third party providers and participants' GPs. All participants will require a baseline blood sample and this will be collected by a research nurse or doctor during the home visit. This home visit will also allow the research nurse or doctor to gather any missing data prior to randomisation, to check that participants are confident in their use of the BP monitor and to highlight to the research team any reason(s) why the participant might not be suitable for continued trial participation. Depending on completeness of the baseline information available and readiness of the IT system, the run-in phase will last a minimum of 7 days and a maximum of 4 months.

During the run-in phase, each participant's GP will be informed that their patient has consented to participate in the trial and provided with the contact details of the research team should they wish to discuss their patient's participation further.

During the run-in phase, participants will be asked to measure their BP and pulse once a day. A mean value of all day-time measurements over a week will be used as the baseline home BP. To estimate clinic BP values for eligibility assessment, 5 mmHg will be added to mean home BP values.[20]

9.6 Randomisation

Participants who remain eligible and willing to continue with the study will be randomised to one of the two treatment arms A or B. Randomisation will be done by authorised research staff using the trial's concealed randomisation system. This web-based randomisation will be based on a minimisation algorithm according to participants' age categories (≤80 years vs >80 years) and baseline systolic BP categories (<130 mmHg, 130-140 mmHg and >140 mmHg). Once the imbalances of these factors have been estimated as a score, the participant will then be allocated to the group with lowest score using a probability greater 0.75 (thus, aiming to minimise the imbalance between groups in a dynamic way).

Participants not meeting the eligibility criteria for continued study participation and randomisation will be informed with reason given. If necessary, a visit will be arranged to collect trial hardware or participants will be asked to return the devices by post for free.

9.7 Blinding and code-breaking

As a partially blinded trial, the trial management system will restrict access to the information on treatment allocation as much as possible by defining different roles, data access and un-blinding procedures.

Participants: participants will be informed of the type of medication change that they have been allocated to (no change, reduction in medicines, increase in medicines) but not the actual treatment arm.

Non-study clinical staff: Patients' usual care team, such as GPs and hospital doctors or nurses will be able to obtain information about participants' treatment plan but not the actual randomised treatment allocation.

Trial personnel following patients for adherence to the protocol: Trained staff involved in participant recruitment will have access to participants' treatment plan to guide the implementation of drug changes over time, but not the actual randomised treatment allocation.

Trial personnel following patients for detection of study outcomes: These members of the study team will be provided restricted access and will be blinded to treatment allocation. This blinding will ensure that the risk of bias is kept to a minimum.

Trial statisticians: All statistical analyses will be pre-specified and treatment allocation will only be made available to statisticians at the final stage of analyses.

With the above procedures in place, there is no need for 'code-breaking' for individual participants during the trial. Non-study clinical staff and trial personnel following patients for adherence to the allocated treatment will be blinded to randomised treatment allocation but not management plan, which means that safety monitoring will focus on participants' medication use and BP as opposed to the randomised treatment allocation (see section 10 SAFETY REPORTING). At the same time, this partial blinding ensures the risk of bias is kept to a minimum.

9.8 Description of study interventions and comparators

A difference in two antihypertensive drugs between treatment arms will be targeted, in which case approximately a 10 mmHg difference in SBP can be expected. Aiming for a minimum difference in intensity of BP lowering treatment and a minimum SBP treatment difference between trial arms has the advantage that participants with a wide range of pre-randomisation SBP values are able to be included. This also obviates the need for a single SBP target which would be difficult to achieve across all baseline SBP groups.

9.8.1 Randomisation arms

Participants eligible for the trial will be randomised to one of two arms:

- **Arm A (more antihypertensive drugs)**: up to two more classes of antihypertensive drugs, vs
- **Arm B (fewer antihypertensive drugs)**: up to two fewer classes of antihypertensive drugs.

As a pragmatic trial, ATEMPT will include participants with different background antihypertensive treatments and a wide range of baseline BP. ATEMPT is not a trial of specific types of medications or a

particular BP target, but rather a trial of "more" versus "less" antihypertensive treatment. To reduce the risk of Type 2 error, a minimum difference of 2 drug classes between treatment arms A and B must be achievable.

9.8.2 Antihypertensive drugs

Use of once-daily preparations of antihypertensive agents will be encouraged unless alternative dosing frequency is indicated/necessary. One or more medications from the classes of agents below will be prescribed by the study personnel and intended for use in managing participants in both randomisation groups to achieve study goals.

The selection of antihypertensive agents will follow the order below according to the most recent European Society of Hypertension guidelines:[19]:

1. Angiotensin converting enzyme (ACE)-inhibitors or angiotensin receptor blockers (ARBs);
2. Thiazide-type diuretics;
3. Dihydropyridine calcium-channel blockers (CCB);
4. Potassium-sparing diuretics (spironolactone);
5. Beta-blockers;
6. Alpha1-receptor blockers.

In treatment arm A, at each assessment point one drug not already in use may be added following the ordered list above (1 to 6), after checking for known intolerances, drug interactions and contra-indications (see Table below). Based on evidence that a combination of multiple drugs at low or moderate dose are preferred to full up-titration of single drugs,[14,31] there will be a maximum of one up-titration for each newly allocated drug (aiming for a half of the daily recommended dose).

In treatment arm B, at each assessment point one drug may be removed (or reduced in dose) following the reverse order in the list above (6 to 1), discounting any drugs that might have been prescribed for other compelling indications as per Table below.

Drug class	Absolute contra-indications	Relative contra-indications
**Diuretics (thiazides/thiazide-like, e.g. chlorthalidone and indapamide)**	Gout	Metabolic syndrome<br>Glucose intolerance<br>Hypercalcaemia<br>Hypokalaemia<br>Hyponatraemia
**Beta-blockers**	Asthma<br>Any high-grade sinoatrial or atrioventricular block<br>Bradycardia (heart rate <60 beats per min)	Metabolic syndrome<br>Glucose intolerance
**Dihydropyridine calcium channel blockers**		Tachyarrhythmia<br>Heart failure with reduced ejection fraction NYHA class III or IV

		Pre-existing severe leg oedema
**Non-dihydropyridine calcium channel blockers**	Any high-grade sinoatrial or atrioventricular block<br>Severe LV dysfunction (LV ejection fraction <40%)<br>Bradycardia (heart rate <60 beats per min)	Constipation
**Angiotensin-converting enzyme inhibitors**	Previous angioneurotic oedema<br>Hyperkalaemia (potassium >5.5 mmol/L)<br>Bilateral renal artery stenosis	
**Angiotensin receptor blockers**	Hyperkalaemia (potassium >5.5 mmol/L)<br>Bilateral renal artery stenosis	
**Potassium-sparing diuretics (Spironolactone)**	Acute renal insufficiency<br>Hyperkalaemia (potassium >5.5 mmol/L)	
**Alpha1-receptor blockers**	Orthostatic hypotension	

Table below summarises drugs used for indications other than hypertension, which should not be stopped in any of the randomised arms. The information about such diseases will be captured through the EHR and direct questions to participants. If deemed appropriate, chart review will be conducted and GPs contacted.

Clinical indication	Drugs
**Heart failure**	Beta-blockers, Angiotensin-converting enzyme inhibitors or angiotensin receptor blockers, mineralocorticoid receptor antagonists
**Atrial fibrillation**	Non-dihydropyridine calcium channel blockers, beta-blockers
**Recent (<12 months) myocardial infarction or angina**	Beta-blockers
**Benign Prostatic Hyperplasia**	Alpha1-receptor blockers

9.8.3 Stratified content of the intervention and comparator

An IT-enabled algorithm will be used to randomly allocate participants to treatment arm A or B (while balancing confounding variables age and baseline BP through minimisation).

Depending on participants' SBP during run-in and number of antihypertensive drug classes (excluding those required for other indications), the trial management software will then deterministically group participants into one of three treatment strata. The stratum allocation occurs after randomisation and is

purely a mean to guide implementation of the randomised allocation. All randomised comparisons will be between group A and B as a whole (with strata functioning as subgroups). The management principle for drug change within each stratum is shown below.

	**Randomised group allocation**	
	**Arm A**	**Arm B**
Stratum 1	Add 2 new drugs	No change
Stratum 2	Add 1 new drug	Stop 1 drug
Stratum 3	No change	Stop 2 drugs

Exact rules used by the software for management of medication changes are shown in the table below.

Estimated clinic SBP (mmHg)*	Randomisation stratum	Number of BP drugs (not for any other essential indications)	Target change in number of BP drugs		Final number of BP drugs		Difference in number of BP drugs
			Treatment Arm A	Treatment Arm B	Treatment Arm A	Treatment Arm B	
115-124	3	2	0	-2	2	0	2
115-124	3	3	0	-2	3	1	2
115-124	3	4	0	-2	4	2	2
115-124	2	1	1	-1	2	0	2
125-134	3	3	0	-2	3	1	2
125-134	3	4	0	-2	4	2	2
125-134	1	0	2	0	2	0	2
125-134	2	1	1	-1	2	0	2
125-134	2	2	1	-1	3	1	2
135-144	3	3	0	-2	3	1	2
135-144	3	4	0	-2	4	2	2
135-144	1	0	2	0	2	0	2
135-144	1	1	2	0	3	1	2
135-144	2	2	1	-1	3	1	2
145-154	3	4	0	-2	4	2	2
145-154	1	0	2	0	2	0	2
145-154	1	1	2	0	3	1	2
145-154	2	2	1	-1	3	1	2
145-154	2	3	1	-1	4	2	2
155-164	1	0	2	0	2	0	2
155-164	1	1	2	0	3	1	2
155-164	1	2	2	0	4	2	2

*Calculated by adding 5mmHg to the average of all home monitored SBP during run-in.

9.9 Communication and delivery of prescription changes, follow-up for BP and safety monitoring

During run-in, participant's GP will be contacted to inform them about the planned randomisation of the individual into the study and the potential implications of this on future care. They will be informed that depending on treatment allocation, there might be the need for change in type and number of anti-hypertensives. They will also be informed that any additional prescriptions will be done by the trial staff, with no additional costs to the GPs. If there is the need for stopping of a prescription, they and the participant will be informed of this. Procedures are in place to avoid miscommunication and harms to the patient. They will be offered to seek further information from the study team if necessary.

Prescription changes and monitoring will be carried out as follows.

After randomisation, participants will be informed via the participant portal and via their chosen method of communication regarding any changes in their medication (stratum, but not the actual treatment arm). Participants will be asked to confirm via the participant portal that they understand their new regimen and reminded of the freephone telephone number and email address to contact the trial team if they have any questions or concerns. If necessary, an automated text message or email will ask participants to log in to the participant portal to acknowledge that they have understood the drug regimen they should follow.

When there are no planned changes to treatment, the participant will be informed to continue usual treatment until further notice. No additional communication will be made with the GP at this stage. Subsequent non-study changes to medications will be monitored through acquisition of prescription changes from the on-line pharmacy on approximately 4-weekly intervals. Additionally, participants are advised to report any changes to their medications as soon as possible.

Where treatment allocation requires stopping of medications, the participant will be informed of this and again asked to confirm that they understand this. Depending on the nature of the change, they might be asked to stop treatment immediately or if a stepwise dose reduction is required, they will be informed that their GP will be asked to initiate the change. In either scenario, the GP will be informed to make the change in prescription. Confirmation of the change will be assessed through 4-weekly updates of the participant's prescription list from the on-line pharmacy and from participants directly.

Where treatment allocation requires addition of medications, the participant will be informed of this with an explanation that the study team will issue a private prescription for the new drug, with an expected delivery date. Medication issuing and postal delivery will be done by the on-line pharmacy typically within 7 days. The change in treatment will be communicated to the participant's GP by post/email. Participants will also be encouraged to report changes to medications, changes in wellbeing or adverse events, as soon as they occur.

The above procedures will be repeated for all randomised participants approximately every 4 weeks until the end of the study. Assessment of the need for change in drug and prescription will be done by trial clinicians who are not be blinded to the patient's treatment allocation and are able to review all relevant

clinical information for making decision (e.g., tolerability issues, previous allergies, BP trends, and communications from GPs and online pharmacy). A clinical management SOP will standardise the process for decision making and clinical safety monitoring.

If clinically indicated, additional assessments will be scheduled. These could include a telephone call to the participant to check for tolerability and clarify any other issues, and occasional referral for safety blood check. Throughout the follow up period, participants will be asked to provide at least weekly BP and pulse readings, unless instructed otherwise.

9.10 Length of Follow Up Period

The length of time that participants remain in follow up will vary depending on the point at which they join the trial. Those joining early will be followed up for a longer period than those joining the trial in the latter stages. The median follow up time is expected to be about 6 months.

This is a pilot study and therefore we do not require a minimum number of follow up visits to be completed by each participant. Instead we plan to obtain as much follow up data as possible from each individual participant during the active phase of the study in order to inform the planning of an adequately powered larger main trial.

9.11 Sample Handling

For safety monitoring and to guide treatment as per clinical practice guidelines, a blood test will be done before randomisation. This will include the assessment of electrolytes and renal function. All baseline samples will be collected by a research nurse during a home visit. Additional blood samples might be required during follow-up and these will be collected by a trained member of the research team. All blood tests will be sent to a central laboratory using pre-paid postage sample collection kits which comply with current Royal Mail requirements. Results will be sent electronically to the trial team.

9.12 Equipment delivery and set-up

During the initial self-registration, participants willing to enter the study-run will be asked about their willingness and capacity to monitor their own BP and pulse. Those who own a certified device are encouraged to start using the monitor on a daily basis. Those who do not own a certified device will be given a study device during the initial home visit by the study nurse. During this visit, the technique for using the equipment and recording of the findings will also be checked.

9.13 Follow-up for recording of adverse events, patient wellbeing and other study outcomes

Participants will be encouraged to report any changes in their wellbeing or adverse events at any point during the follow up period via the trial portal or directly to the research team via the freephone line if they would prefer. In addition, study personnel who are blinded to treatment allocation will contact the patient every 3 months to capture information about adverse events, patient wellbeing and other study outcomes. Finally, information about adverse events and repeat prescriptions will be obtained periodically from linkage with national registers and the on-line pharmacy, respectively. For quality assurance, home visits might be arranged for a random number of participants to check usability and validity of information provided.

9.14 Final Visit

All willing participants will be followed up until the last participant has been randomised and has reached the stage where no further medication changes are indicated. Participants will be informed when their participation in the trial will end and arrangements will be made to collect the BP monitoring equipment.

Before trial participation ends, each participant's GPs will be informed and provided with the participant's current anti-hypertensive medications. Upon completing the study, the participant will be returned to the care of their GP who will be able to maintain the current anti-hypertensive medications or amend the prescription as they deem appropriate.

9.15 Early Discontinuation/Withdrawal of Participants

Participants will be free to withdraw from the trial at any point, in which case their GP will be informed of their decision and a list of current BP medications provided to the GP. Participants will be asked to return any equipment that they might hold to the study team and will be offered the opportunity to provide feedback on the trial procedures and equipment as well as to make suggestions for improvement. Participants may also explain why they want to withdraw from the study, but they will not be required to do so. Participants will have the following two options for withdrawal:

1) Participants may withdraw from active follow-up and further communication but allow the study team to continue to access their medical records and any relevant hospital data that is recorded as part of routine standard of care;
2) Participants may withdraw from active follow-up and further communication <u>and</u> withdraw their permission to allow the study team to continue to access their medical records and other relevant hospital data meaning that no further data would be collected after withdrawal.

In addition, the Investigator may discontinue a participant from the trial treatment at any time (but not from trial follow-up) if the Investigator considers it necessary for any reason including, but not limited to:

- Significant non-compliance with treatment regimen or study requirements, which renders the patient at risk for adverse events
- Clinical decision that the patient will be a high risk of adverse events in case trial treatment continues

If a participant withdraws or is withdrawn due to a Serious Adverse Event (SAE), where possible and with the continued consent of the participant (option 1 above), the SAE will be followed up until stabilised or resolved whichever is earlier.

Withdrawal from the trial will not result in exclusion of the previously recorded data for that participant from analysis (to reduce the risk of bias from loss-to-follow-up in an intention-to-treat design), and will not result in exclusion from further follow-up, as described in the protocol.

Withdrawn participants will be replaced if the withdrawal takes place within the planned recruitment period. The type of withdrawal and reason for withdrawal will be recorded in the trial database.

9.16 Definition of End of Study

The end of study is the point at which all the study data has been received for the last participant and all data queries resolved.

10 SAFETY REPORTING

ATEMPT is a trial of different anti-hypertensive management strategies rather than an Investigational Medicinal Product per se. Consequently, it has been categorised by the Medicines and Healthcare products Regulatory Agency (MHRA) as not being a Clinical Trial of an Investigational Medicinal Product (CTIMP). Nonetheless, collection of adverse clinical events will form an important and necessary component of clinical monitoring and management of participants, and they are also necessary for evaluation of the intervention, including any tolerability or safety issues concerning the different treatment strategies. Thus, the following definitions and safety reporting procedures will be followed to facilitate clinical management, ensure participant safety, and systematic collection of outcome information.

10.1 Definition of Serious Adverse Events

A serious adverse event (SAE) is any untoward medical occurrence that:

- results in death
- is life-threatening
- requires inpatient hospitalisation or prolongation of existing hospitalisation
- results in persistent or significant disability/incapacity
- consists of a congenital anomaly or birth defect.

Other ‘important medical events’ will be considered a serious adverse event when, based upon appropriate medical judgement, the event may jeopardise the participant and may require medical or surgical intervention to prevent one of the outcomes listed above.
NOTE: The term "life-threatening" in the definition of "serious" refers to an event in which the participant was at risk of death at the time of the event; it does not refer to an event which hypothetically might have caused death if it were more severe.

10.2 Reporting Procedures for Serious Adverse Events

All relevant SAEs will be reported to relevant parties (including the Chairman of the Data Monitoring Committee (DMC), regulatory authorities, appropriate ethics committees and trial sponsor) on an annual basis.

Relevant SAEs are those that could inform about the safety of treatment strategies and include cardiovascular outcomes (stroke, TIA, myocardial infarction, coronary procedures, atrial fibrillation) or safety outcomes (renal failure, hypotensive episodes with falls or fracture, delirium/confusion, renal failure and death, as collected from central databases and participant questionnaires. All relevant SAEs will be followed up until resolution, or participant completion in the study, whichever is earlier.

10.3 Non-Serious Adverse Events

Non-serious adverse events will not be recorded routinely, unless such events are thought to be related to the study treatment by participants or the Investigator. Participants will be encouraged to report any symptoms or events felt to be related to treatment. In addition, at regular assessment points, participants will specifically be asked by blinded study personnel about the following events, irrespective of their seriousness:

- Falls or fracture
- Dizziness or fainting
- Loss of consciousness or collapse
- Confusion

For all participants, the BP measurements received via the trial portal will be automatically summarised, with substantial changes between measurements flagged for review by the study team. An SOP defining the action required for the potential range of abnormal observations will be implemented prior to the start of the trial.

10.4 Development Safety Update Reports and the Role of DMC

A Data Monitoring Committee (DMC) will be established and will meet every 12 months, usually before the Trial Oversight Committee (TOC) meetings. An unblinded analysis of all adverse events and other study outcomes will be provided in strict confidence to the Chairman of the DMC. In light of these analyses and any other information considered relevant, the DMC will advise the TOC if, in their view, the randomised comparisons in the study have provided **both** (i) “proof beyond reasonable doubt” that for all, or some specific types of, participants prolonging the trial intervention is clearly harmful; **and** (ii) evidence that might reasonably be expected to influence materially the patient management of many clinicians who are already aware of the results of other trials. Appropriate criteria of proof beyond reasonable doubt cannot be specified precisely, but in general a difference of at least 2 standard deviations in an interim analysis of mortality or major morbidity would be needed to justify halting, or modifying, the study prematurely. This criterion has the practical advantage that the exact number of interim analyses is of little importance. With the DMC advice, the TOC can then decide whether to modify the study, or to seek additional data. Unless this happens, the CI, Investigators, study participants, and all study staff (except those who provide the confidential analyses to the DMC) will remain blind to the comparative results until the end of the study. Role of the DMC and decision guidance will be specified in a DMC charter.

11 STATISTICS AND ANALYSIS

11.1 Statistical Analysis Plan (SAP)

The statistical aspects of the study are summarised here with details to be fully described in a Statistical Analysis Plan (SAP) that will be available before data lock and unblinding takes place.

11.2 Description of the Statistical Methods

Measurement of outcomes and their frequency has been described in section 6.2 Outcome measures and assessment. Randomised comparisons will be by intention to treat (ITT). As for BP as the primary outcome of the trial, analyses will be two-staged, whereby possible multiple measurements during a single day will be averaged (with some plausibility checks) and then a mixed effects model for repeated

measures will be used to compare the difference between treatment groups. This method has the advantage of using all information available and handling the correlation between repeated measures in the context of variable frequency of measurement by each individual. As complementary measures, summary measures of BP (time-weighted mean) for different periods of follow-up will be reported, together with its 95% confidence interval and compared using a linear model with and without adjustment for stratification variables.[32]

Analysis of the feasibility outcomes will be descriptive.

11.3 Sample Size Determination

As a pilot study, sampling aims to include a diverse group of participants to gain insight into feasibility and tolerability of remote BP management, and the usability of the system and procedures. We will aim to oversample very old (over 80 years-old) participants since this is the age group in whom BP management is more uncertain, and the entry criteria will ensure that our sample includes participants with multimorbidity. Although we understand that lack of IT competency may limit access to this trial, we will try to involve carers whenever needed, if participants are happy for their carers to be involved.

Prior evidence on range and distribution of SBP in a similar setting to ATEMPT is currently unavailable. Therefore, sample size estimations are based on our best guess at the design stage. However, we intend to perform interim blinded analyses of SBP to refine the sample size in the course of the trial. The plot below shows the power (y-axis) achievable with a range of mean differences (x-axis) and sample sizes (curves) using a simple t-test and assuming a standard deviation of around 20 mmHg. A total sample size of 200 patients would provide 80% power to detect a mean difference of 8 mmHg or more than 90% power for a mean difference of 10 mmHg.

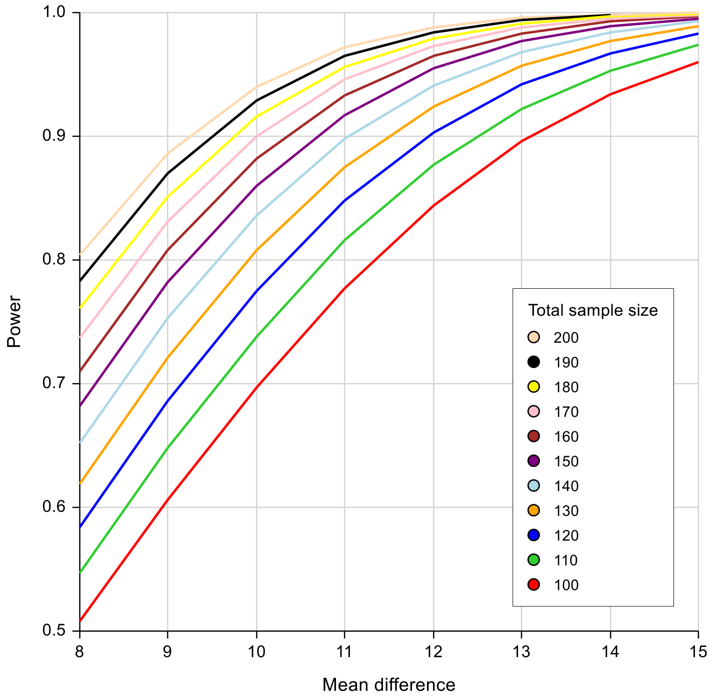

Given that we will use a repeated-measure analysis where every patient with at least one data point will be included, we expect a negligible number of patients to be excluded from the analysis due to missing data. In addition, the use of a repeated-measure model should reduce variability and increase the power calculated under the assumption of a simple t-test.

A sample size of 200 participants should also provide enough information to assess the feasibility of the proposed study.

11.4 Analysis populations

All participants as recruited, for randomisation comparisons, all participants as randomised.

11.5 The Level of Statistical Significance

All final analyses will be conducted using a two-sided type-I error rate of 5% with potential adjustments to the nominal level depending on the number and nature of unblinded interim analyses.

12 DATA MANAGEMENT

The plan for the data management of the study is outlined below.

12.1 Source Data

Source data in ATEMPT will originate from different authorised data originators and are defined as the source where they have been first recorded. Authorised data originators include, but are not limited to:

- Participants and their authorised carers
- Study clinical Investigator(s) and their delegated study staff
- Electronic health records, including laboratory test results and e-prescription lists
- Blood pressure measurement devices
- Any other written or electronic correspondence of relevance.

All direct eCRF entries by authorised users will be considered source data elements (symptoms, questionnaires, blood pressure values entered onto the trial portal, etc.). By contrast, if steps are required to process the data from EHR or devices (e.g., summarising multiple measurements into one or phenomapping of diagnosis from EHR), the raw data from the device or EHR will be the source.

All source data will be stored safely in confidential conditions and are available for review and auditing. On all study-specific documents, other than the signed consent and data used for clinical management and communication with non-study doctors, pharmacists or nurses, the participant will be referred to by the study participant number/code, not by name.

12.2 Access to Data

Direct access will be granted to authorised representatives from the Sponsor and host institution for monitoring and/or audit of the study to ensure compliance with regulations.

12.3 The Clinical Trial Management System

The ATEMPT clinical trial management system will consist of a custom-written and validated web-based, software platform, which can integrate with external devices and applications, where appropriate. The

system comprises of an end-to-end solution for managing all the data and processes required to conduct the study. There are three main system components: the Trial Portal ('Ivy' for the trial management team), the Participant Portal ('Aspen' for study participants) and the API (for secure data exchange and system integration).

12.4 Data Processing

Trial data will be captured by authorised data originators (including participants), imported into the platform and recorded on eCRFs. Trial data collected via trial and participant portals will be securely transmitted to the trial database servers, hosted in a high security environment. No data will be stored within the Web Applications Server. The Trial Portal will allow access to patient identifiable information only to authorised users. Data from external sources (e.g., hospital admission episodes or death registry) will be sent periodically to the research database (ATEMPT database) for storage and analysis, without the knowledge of intervention allocation.

The research database (ATEMPT database) will hold data from study participants. A separate database (Pre-screening database) will keep data from potential participants retrieved from third parties and will only be used for participant invitation.

Participant identifiable data provided by third parties about potential participants will be stored in the pre-screening database until individuals consent to participate in the study when some of their details will be copied to the ATEMPT database. Name, email address and/or phone number will be collected directly from potential participants prior to consent (and stored in the ATEMPT database) and if the potential participant fails to provide consent to participate within 30 days or they are unable to participate due to an exclusion criterion being met, their details will be deleted from the ATEMPT database.

For the trial team, access to the Trial Portal will be granted according to access level which will be dependent on the role of the user. Authentication will be carried out by a username and a password.

For participants, access to the Participant Portal will require a unique access code provided by the trial team.

The Trial Portal and Participant Portal will make use of electronic prompts, flags, and data quality checks in the eCRF to minimize errors and omissions during data entry. Prompts will be designed to alert the data originator to missing data, inconsistencies and implausible values (e.g., date out of range). In addition to entering their BP readings the participants will be able to update their contact details and view the BP readings they have submitted.

Any changes to data will be logged automatically within the Trial and Participant portals and will be linked to the user. Modified and/or corrected data elements will have data element identifiers that reflect the date, time, originator and reason for the change, and will not obscure previous entries. A field will be provided allowing originators to describe the reason for the change (e.g., transcription error). Automatic transmissions will have traceability and controls via the audit trail to reflect the reason for the change.

All study documents will be stored securely and only accessible for study staff and authorised personnel. All data transfers will be cryptographically secured, and all data will be stored securely. Data in the ATEMPT database will be de-identified at the end of the study and the data stored for at least 5 years after the study has ended. The data will be stored and archived in accordance with the University of Oxford data management policies. Study and clinical staff access will be restricted according to their role in the study.

The participants will be identified by a unique participant study number in any database. The name and any other identifying detail will NOT be accessible to unauthorised team members.

Within the consent process, participants will be given the option to be approached for future research, in which case data management will be fully compliant with GDPR. The consent form will be retained as the basis for future approach. Contact details will be held securely, separately from the research data, and kept updated.

13 QUALITY ASSURANCE PROCEDURES

The study may be monitored, or audited in accordance with the current approved protocol, GCP, relevant regulations and standard operating procedures.

13.1 Risk assessment

A risk assessment and monitoring plan will be prepared before the study opens and will be reviewed as necessary over the course of the study to reflect significant changes to the protocol or outcomes of monitoring activities.

13.2 Study monitoring

As a non-CTIMP, GCP monitoring will not be required for this study.

13.3 Study committees

13.3.1 Trial Management Group

The Trial Management Group (TMG) will be responsible for the day-to-day management of the trial and will meet regularly (at least monthly) to discuss the planning and progress of the trial. The TMG will be made up of named individuals from the trial team and chaired by the CI.

13.3.2 Trial Steering Committee

The ATEMPT trial will be overseen by Trial Steering Committee (TSC), whose role will be to guide the research agenda, advise on the plan of investigation, and monitor the execution of the project of behalf of the project sponsor and project funder. The TSC will meet in person every twelve months with some email and telephone communication in between. The TSC will be composed of an independent chair, members of a patient group or individuals able to contribute to the wider public perspective and named representatives from the TMG, including the chief investigator.

13.3.3 Data Monitoring Committee

A Data Monitoring Committee (DMC) will be responsible for monitoring the trial data and the continued safety of research participants and will be the only committee permitted access to unblinded comparative data during the conduct of the trial. The DMC will meet in person every 12 months and the meetings will be timed to coincide with TSC meetings such that reports and findings can be made available for the TOC to discuss. The DMC will be composed of an independent chair, an independent statistician and the chair of the TSC.

14 PROTOCOL DEVIATIONS

A study related deviation is a departure from the ethically approved study protocol or other study document or process (e.g. consent process) or from Good Clinical Practice (GCP) or any applicable regulatory requirements. Any deviations from the protocol will be documented in a protocol deviation form and filed in the trial master file.

15 SERIOUS BREACHES

A "serious breach" is a breach of the protocol or of the conditions or principles of Good Clinical Practice which is likely to affect to a significant degree

(a) the safety or physical or mental integrity of the trial participants; or
(b) the scientific value of the research.

In the event that a serious breach is suspected the Sponsor must be contacted within 1 working day. In collaboration with the CI, the serious breach will be reviewed by the Sponsor and, if appropriate, the Sponsor will report it to the approving REC committee and the relevant NHS host organisation within seven calendar days.

16 ETHICAL AND REGULATORY CONSIDERATIONS

16.1 Declaration of Helsinki

The Investigator will ensure that this study is conducted in accordance with the principles of the Declaration of Helsinki.

16.2 Guidelines for Good Clinical Practice

The Investigator will ensure that this study is conducted in accordance with relevant regulations and with Good Clinical Practice.

16.3 Approvals

Following Sponsor approval, the protocol, informed consent form, participant information sheet, invitation letters and advertising material will be submitted to the appropriate Research Ethics Committee (REC), and host institutions for written approval.
The Investigator will submit and, where necessary, obtain approval from the above parties for all substantial amendments to the original approved documents.

16.4 Other Ethical Considerations

None.

16.5 Reporting

The CI shall submit once a year throughout the study, or on request, an Annual Progress report to the REC, host organisation, Sponsor and funder. In addition, an End of Study notification and final report will be submitted to the same parties.

16.6 Participant Confidentiality

The study will comply with the General Data Protection Regulation (GDPR) and Data Protection Act 2018, which require data to be de-identified as soon as it is practical to do so. The processing of the personal data of participants will be minimised by making use of a unique participant study number only on study documents and any electronic database(s). All documents will be stored securely and only accessible by study staff and authorised personnel. The study staff will safeguard the privacy of participants' personal data. The study will comply with the specific requirements of the University of Oxford (Data Protection Checklist https://researchsupport.admin.ox.ac.uk/policy/data/checklist; and Practical Considerations https://researchsupport.admin.ox.ac.uk/policy/data/practical).

16.7 Expenses and Benefits

We do not expect participants to incur any expenses by taking part in this study.

17 FINANCE AND INSURANCE

17.1 Funding

The trial is funded by research grants from the NIHR Oxford BRC and the Oxford Martin School.

17.2 Insurance

The University has a specialist insurance policy in place which would operate in the event of any participant suffering harm as a result of their involvement in the research (Newline Underwriting Management Ltd, at Lloyd's of London).

17.3 Contractual arrangements

Appropriate contractual arrangements will be put in place with all third parties.

18 PUBLICATION POLICY

The Trial Management Group will coordinate and take responsibility for all publications. Papers will be written and authored by selected members in the Trial Management Group, in the name of the ATEMPT Collaborative Group, with individual members named personally at the end of the report (or, to comply with journal requirements in web-based material posted with the report). The Investigator will be involved in reviewing drafts of the manuscripts, abstracts, press releases and any other publications arising from the study. Authorship will be determined in accordance with the ICMJE guidelines and other contributors will be acknowledged. The Trial Management Group will aim to publish the trial findings as open access to ensure that it is widely available. Authors will acknowledge that role of the funders in any publication arising from the study. All publications and release of data will be compliant with relevant regulations and recommendations on transparency in clinical research.

19 DEVELOPMENT OF A NEW PRODUCT/ PROCESS OR THE GENERATION OF INTELLECTUAL PROPERTY

Ownership of IP generated by employees of the University vests in the University. The protection and exploitation of any new IP is managed by the University's technology transfer office, Oxford University Innovations.

20 ARCHIVING

Once all study-related activity has ceased, all study documentation including essential documents, source documentation and CRFs will be archived according to departmental guidelines. All study documentation will be retained for at least 5 years after the completion of study-related activities in accordance with the requirements of the University of Oxford. Hard copy documentation will be filed securely in a lockable room or cupboard with access restricted to authorised personnel only. All electronic data including study documentation and any databases holding trial information will be backed up onto a secure server with access restricted to authorised personnel only.

21 REFERENCES

1. Ettehad D, Emdin CA, Kiran A, et al. Blood pressure lowering for prevention of cardiovascular disease and death: a systematic review and meta-analysis. *Lancet* 2016; **387**(10022): 957-67.
2. Collerton J, Davies K, Jagger C, et al. Health and disease in 85 year olds: baseline findings from the Newcastle 85+ cohort study. *BMJ (Clinical research ed)* 2009; **339**: b4904.
3. Pinho-Gomes AC, Rahimi K. Blood pressure management in the elderly: the need for more randomised evidence. *Heart* 2019; **105**(14): 1055-6.
4. Berlowitz DR, Foy CG, Kazis LE, et al. Effect of Intensive Blood-Pressure Treatment on Patient-Reported Outcomes. *The New England journal of medicine* 2017; **377**(8): 733-44.
5. Yarnall AJ, Sayer AA, Clegg A, Rockwood K, Parker S, Hindle JV. New horizons in multimorbidity in older adults. *Age Ageing* 2017; **46**(6): 882-8.
6. Santoni G, Calderon-Larranaga A, Vetrano DL, Welmer AK, Orsini N, Fratiglioni L. Geriatric health charts for individual assessment and prediction of care needs: a population-based prospective study. *J Gerontol A Biol Sci Med Sci* 2018.
7. Todd OM, Wilkinson C, Hale M, et al. Is the association between blood pressure and mortality in older adults different with frailty? A systematic review and meta-analysis. *Age and ageing* 2019.
8. Williamson JD, Supiano MA, Applegate WB, et al. Intensive vs Standard Blood Pressure Control and Cardiovascular Disease Outcomes in Adults Aged ≥75 Years: A Randomized Clinical Trial. *Jama* 2016; **315**(24): 2673-82.
9. Warwick J, Falaschetti E, Rockwood K, et al. No evidence that frailty modifies the positive impact of antihypertensive treatment in very elderly people: an investigation of the impact of frailty upon treatment effect in the HYpertension in the Very Elderly Trial (HYVET) study, a double-blind, placebo-controlled study of antihypertensives in people with hypertension aged 80 and over. *BMC Med* 2015; **13**: 78.
10. Turnbull F, Neal B, Ninomiya T, et al. Effects of different regimens to lower blood pressure on major cardiovascular events in older and younger adults: meta-analysis of randomised trials. *Bmj* 2008; **336**(7653): 1121-3.
11. Aguayo GA, Vaillant MT, Donneau AF, et al. Comparative analysis of the association between 35 frailty scores and cardiovascular events, cancer, and total mortality in an elderly general population in England: An observational study. *PLoS Med* 2018; **15**(3): e1002543.
12. Karmali KN, Lloyd-Jones DM, van der Leeuw J, et al. Blood pressure-lowering treatment strategies based on cardiovascular risk versus blood pressure: A meta-analysis of individual participant data. *PLoS Med* 2018; **15**(3): e1002538.
13. Davies EA, O'Mahony MS. Adverse drug reactions in special populations - the elderly. *Br J Clin Pharmacol* 2015; **80**(4): 796-807.
14. Benetos A, Petrovic M, Strandberg T. Hypertension Management in Older and Frail Older Patients. *Circulation research* 2019; **124**(7): 1045-60.
15. Streit S, Poortvliet RKE, Elzen W, Blom JW, Gussekloo J. Systolic Blood Pressure and Cognitive Decline in Older Adults With Hypertension. *Ann Fam Med* 2019; **17**(2): 100-7.
16. Ravindrarajah R, Hazra NC, Hamada S, et al. Systolic Blood Pressure Trajectory, Frailty, and All-Cause Mortality >80 Years of Age: Cohort Study Using Electronic Health Records. *Circulation* 2017; **135**(24): 2357-68.
17. Delgado J, Bowman K, Ble A, et al. Blood Pressure Trajectories in the 20 Years Before Death. *JAMA Intern Med* 2018; **178**(1): 93-9.
18. Whelton PK, Carey RM, Aronow WS, et al. 2017 ACC/AHA/AAPA/ABC/ACPM/AGS/APhA/ASH/ASPC/NMA/PCNA Guideline for the Prevention, Detection, Evaluation, and Management of High Blood Pressure in Adults: A Report of the American College of Cardiology/American Heart Association Task Force on Clinical Practice Guidelines. *Hypertension (Dallas, Tex : 1979)* 2018; **71**(6): e13-e115.
19. Williams B, Mancia G, Spiering W, et al. 2018 ESC/ESH Guidelines for the management of arterial hypertension. *European Heart Journal* 2018; **39**(33): 3021-104.

20. NICE. Hypertension in adults: diagnosis and management. UK: National Institute for Health and Care Excellence, 2019.
21. NICE. Multimorbidity: the assessment, prioritisation and management of care for people with commonly occurring multimorbidity. NICE guideline [NG56]. UK: National Institute for Health and Clinical Excellence; 2016.
22. Blood Pressure System Leadership Board. Tackling high blood pressure: an update. London, UK: Public Health England, 2015.
23. Lockett J, Sauma S, Radziszewska B, Bernard MA. Adequacy of Inclusion of Older Adults in NIH-Funded Phase III Clinical Trials. *J Am Geriatr Soc* 2019; **67**(2): 218-22.
24. Bobrie G, Chatellier G, Genes N, et al. Cardiovascular prognosis of "masked hypertension" detected by blood pressure self-measurement in elderly treated hypertensive patients. *Jama* 2004; **291**(11): 1342-9.
25. Triantafyllidis A, Velardo C, Chantler T, et al. A personalised mobile-based home monitoring system for heart failure: The SUPPORT-HF Study. *International journal of medical informatics* 2015; **84**(10): 743-53.
26. Rahimi K, Velardo C, Triantafyllidis A, et al. A user-centred home monitoring and self-management system for patients with heart failure: a multicentre cohort study. *European heart journal Quality of care & clinical outcomes* 2015; **1**(2): 66-71.
27. Wang J, Shi X, Ma C, et al. Visit-to-visit blood pressure variability is a risk factor for all-cause mortality and cardiovascular disease: a systematic review and meta-analysis. *J Hypertens* 2017; **35**(1): 10-7.
28. Salam A, Atkins E, Sundstrom J, et al. Effects of blood pressure lowering on cardiovascular events, in the context of regression to the mean: a systematic review of randomized trials. *J Hypertens* 2019; **37**(1): 16-23.
29. Sega R, Facchetti R, Bombelli M, et al. Prognostic value of ambulatory and home blood pressures compared with office blood pressure in the general population: follow-up results from the Pressioni Arteriose Monitorate e Loro Associazioni (PAMELA) study. *Circulation* 2005; **111**(14): 1777-83.
30. Aung T, Haynes R, Barton J, et al. Cost-effective recruitment methods for a large randomised trial in people with diabetes: A Study of Cardiovascular Events iN Diabetes (ASCEND). *Trials* 2016; **17**(1): 286.
31. Chow CK, Thakkar J, Bennett A, et al. Quarter-dose quadruple combination therapy for initial treatment of hypertension: placebo-controlled, crossover, randomised trial and systematic review. *Lancet (London, England)* 2017; **389**(10073): 1035-42.
32. Andersen SW, Millen BA. On the practical application of mixed effects models for repeated measures to clinical trial data. *Pharm Stat* 2013; **12**(1): 7-16.

22 APPENDIX A: STUDY FLOW CHART

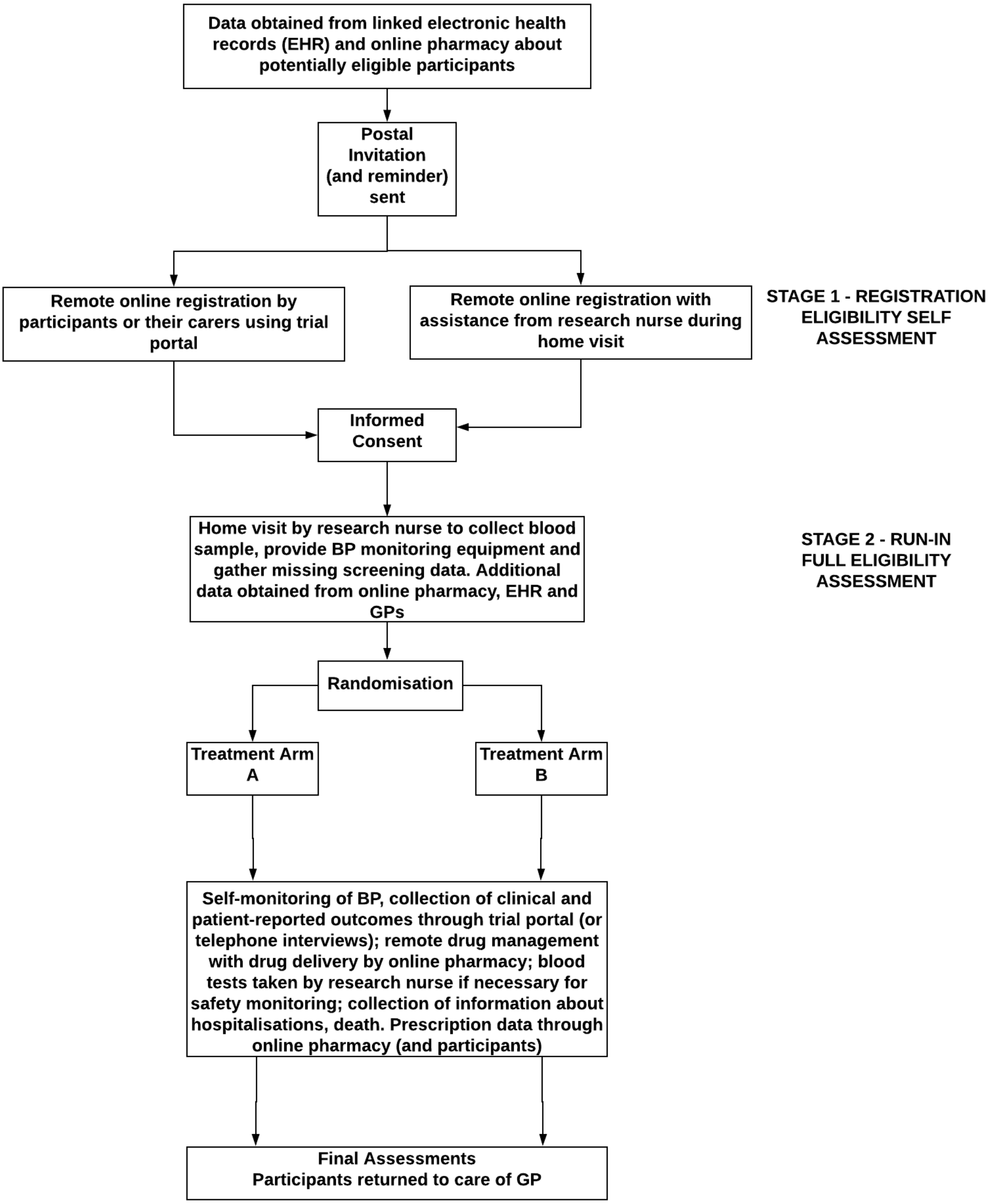

23 APPENDIX B: SCHEDULE OF PROCEDURES

	**Registration and screening assessment**	**Run-in phase** 7 days to 4 months [o]	**Follow-up phase** 6 to 12 months [p]	**Final assessment**
**Self-reports via participant portal[a]**				
Registration[b]	x			
Initial eligibility assessment[b]	x			
e-consent[b]	x			
BP & pulse measurements[c]		x	x	x
Frailty assessment (PRISMA 7)[d]		x	x	x
Quality of life questionnaire [d]		x	x	x
Medication compliance assessment [d]			x	x
Change in medications[e]		x	x	x
Adverse event assessment[e]		x	x	x
**Procedures by study staff**				
Demographics and medical history[f]		x		
Concomitant medications[f]		x		
Cognitive function assessment (T-MoCA) [f,g]		x	x	x
Provide BP monitoring equipment[f,h]		x		
Blood sample[f,j]		x	(x)	(x)
Linkage with on-line pharmacy[k]		x	x	x
Linkage with NHS Digital			x	
GP communications[l]		x	x	x
Final eligibility assessment and randomisation		x		
Treatment review & adjustment[m]			x	
Adverse event assessment and completion of missing information[n]			x	x
Collect equipment				x

(x) Optional assessments

[a] Information will be provided by participants (with assistance from carers, if required) and captured through the online portal.

[b] If requested by participant, research nurse/doctor will schedule a home visit to complete trial registration process and seek consent with all information captured via participant portal.

[c] Recommended frequency of measurement is daily during run-in and weekly after randomisation.

[d] Prompts for completion of questionnaires will be displayed during run-in, and then 3-monthly after randomisation and again at the final assessment.

[e] Prompts for reporting of any change to medication or adverse events will always be displayed when participants log-in to the portal. Participants report might be followed up by a call to complete information.

[f] During home visit by study nurse/doctor.

[g] Assessed over the phone, unless concurrent with a home visit.

[h] If participant does not own their own certified upper-arm BP monitor.

[j] Creatinine, urea, potassium and sodium to assess renal function. During run-in, blood samples will be taken by research nurses performing a home visit. Any subsequent blood samples that are required may

be collected by a trained member of the research team. During the follow up period, up to three further blood samples may be requested to monitor kidney function depending on which antihypertensive medications are prescribed.

[k] To collect information about repeat prescriptions before randomization and then approximately every 4 weeks after randomization (in addition to ad-hoc self-reports by participants).

[l] GPs will be informed about participant's recruitment into the study prior to randomisation. After randomisation, they will be informed of any allocated treatment changes and any actions that they might have to take.

[m] Treatment adjustments to follow the randomised treatment allocation. All trial medication will be ordered through the on-line pharmacy and distributed by post. Stepwise changes to medications will be done approximately every 4 weeks.

[n] Approximately every 3 months participants will receive a call to collect additional or missing information about adverse events and their health status

[o] Length of participation in run-in period dependent on time taken to taken to collect and verify screening information and participant and research team confidence/satisfaction with use of BP monitoring equipment.

[p] Randomised participants will be followed up until the study end unless they choose to withdraw. In practice this will mean that those participants randomised early on may be followed up for 12 months while those randomised later may be followed up for less than 6 months. It is estimated that the median duration of follow up for all participants will be 6 months.

24 APPENDIX C: AMENDMENT HISTORY

Amendment No.	Protocol Version No.	Date issued	Author(s) of changes	Details of Changes made
1.0	3.0	26 Apr 21	K Rahimi<br>D Hedgecott<br>W Turpie	Amendments to trial team and Steering Committee members; Amendment to trial team contact details; Update to trial recruitment period and an extension to the trial duration; Correction to SAE definition; Minor clarifications to trial procedures and participant use of participant portal; Clarification of data processing activities; Addition of new method of recruitment; Updates to Drug Class contraindications tables; Minor corrections to typographical errors.
